# The emerging roles of sphingosine 1-phosphate and SphK1 in cancer resistance: a promising therapeutic target

**DOI:** 10.1186/s12935-024-03221-8

**Published:** 2024-02-28

**Authors:** Samar Sami Alkafaas, Mohamed I. Elsalahaty, Doha F. Ismail, Mustafa Ali Radwan, Sara Samy Elkafas, Samah A. Loutfy, Rami M. Elshazli, Narjes Baazaoui, Ahmed Ezzat Ahmed, Wael Hafez, Mohanad Diab, Mohamed Sakran, Mohamed T. El-Saadony, Khaled A. El-Tarabily, Hani K. Kamal, Mohamed Hessien

**Affiliations:** 1 Molecular Cell Biology Unit, Division of Biochemistry, Department of Chemistry, Faculty of Science, Tanta University, Tanta, 31527 Egypt; 2 Biochemistry Division, Department of Chemistry, Faculty of Science, Tanta University, Tanta, 31527 Egypt; 3 Production Engineering and Mechanical Design Department, Faculty of Engineering, Menofia University, Menofia, Egypt; 4 Faculty of Control System and Robotics, ITMO University, Saint-Petersburg, 197101 Russia; 5 Virology and Immunology Unit, Cancer Biology Department, National Cancer Institute, Cairo University, Cairo, Egypt; 6 Nanotechnology Research Center, British University, Cairo, Egypt; 7 Biochemistry and Molecular Genetics Unit, Department of Basic Sciences, Faculty of Physical Therapy, Horus University—Egypt, New Damietta, 34517 Egypt; 8 Biology Department, College of Sciences and Arts Muhayil Assir, King Khalid University, Abha 61421, Saudi Arabia; 9 Biology Department, College of Science, King Khalid University, Abha 61413, Saudi Arabia; 10 NMC Royal Hospital, 16th Street, 35233 Khalifa, Abu Dhabi, United Arab Emirates; 11 Medical Research Division, Department of Internal Medicine, The National Research Centre, Cairo 11511, Egypt; 12 Burjeel Hospital Abu Dhabi, Abu Dhabi, United Arab Emirates; 13 Biochemistry Department, Faculty of Science, University of Tabuk, Tabuk 47512, Saudi Arabia; 14 Department of Agricultural Microbiology, Faculty of Agriculture, Zagazig University, Zagazig 44511, Egypt; 15 Department of Biology, College of Science, United Arab Emirates University, Al-Ain 15551, United Arab Emirates; 16 Anatomy and Histology, Faculty of Pharmacy, King Abdulaziz University, Jeddah 21589, Saudi Arabia

**Keywords:** Cancer, Recurrence, Chemoresistance, S1P, SphK1, TME

## Abstract

Cancer chemoresistance is a problematic dilemma that significantly restrains numerous cancer management protocols. It can promote cancer recurrence, spreading of cancer, and finally, mortality. Accordingly, enhancing the responsiveness of cancer cells towards chemotherapies could be a vital approach to overcoming cancer chemoresistance. Tumour cells express a high level of sphingosine kinase-1 (SphK1), which acts as a protooncogenic factor and is responsible for the synthesis of sphingosine-1 phosphate (S1P). S1P is released through a Human ATP-binding cassette (ABC) transporter to interact with other phosphosphingolipids components in the interstitial fluid in the tumor microenvironment (TME), provoking communication, progression, invasion, and tumor metastasis. Also, S1P is associated with several impacts, including anti-apoptotic behavior, metastasis, mesenchymal transition (EMT), angiogenesis, and chemotherapy resistance. Recent reports addressed high levels of S1P in several carcinomas, including ovarian, prostate, colorectal, breast, and HCC. Therefore, targeting the S1P/SphK signaling pathway is an emerging therapeutic approach to efficiently attenuate chemoresistance. In this review, we comprehensively discussed S1P functions, metabolism, transport, and signaling. Also, through a bioinformatic framework, we pointed out the alterations of SphK1 gene expression within different cancers with their impact on patient survival, and we demonstrated the protein–protein network of SphK1, elaborating its sparse roles. Furthermore, we made emphasis on different machineries of cancer resistance and the tight link with S1P. We evaluated all publicly available SphK1 inhibitors and their inhibition activity using molecular docking and how SphK1 inhibitors reduce the production of S1P and might reduce chemoresistance, an approach that might be vital in the course of cancer treatment and prognosis.

**Graphical Abstract:**

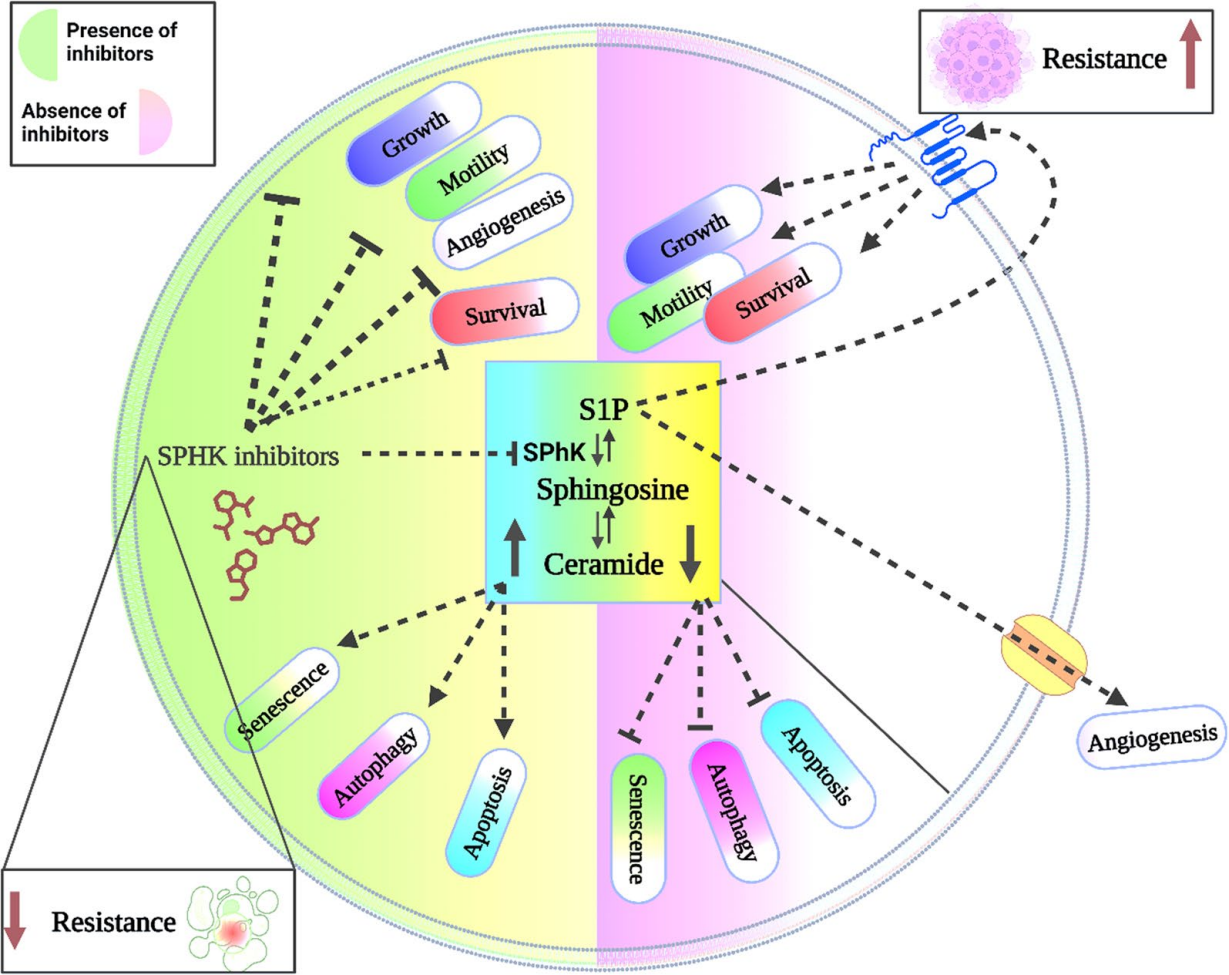

**Supplementary Information:**

The online version contains supplementary material available at 10.1186/s12935-024-03221-8.

## Introduction

Chemotherapy is considered a reliable approach that is utilized against cancer, and recently, it became part of the most common promising treatment protocols against cancer [1–3]. Unfortunately, the expected results weren’t satisfying. Malignant cancer cells are characterized by significantly numerous genotypes and phenotypes as compared to normal cells, which reflect dynamic changes of the genome as well as uncontrolled growth [1, 4–6]. Furthermore, the progression of carcinoma disrupts the biological machinery of neighboring healthy cells through invasion and metastasis. Cancer treatment has recently become more sophisticated, but still, no chemotherapy has an optimal distractive effect against metastatic cells. Chemotherapy fails to treat the majority of cancer patients due to provoked resistance against chemotherapy that subsequently causes cancer cell invasion as well as progression to metastasis [7].

After chemotherapy treatment for a long period, cancer cells gradually become resistant to almost all chemotherapeutic drugs through different machineries, including intrinsic or extrinsic machineries, causing a breakdown in cancer treatment. Intrinsic resistance originates from the properties of cancer cells or tissues themselves that naturally reduce the effectiveness of given cancer chemotherapeutics. Conversely, extrinsic resistance may be acquired and developed during tumor treatment. Cancer cells are initially sensitive during treatment with chemotherapy, while during treatment, responsiveness has deteriorated, and the promising therapeutic effects are attenuated [8]. There are several factors in cancer cell resistance, such as genetic factors and regulatory RNAs, including micro RNAs (miRNAs) as well as long noncoding RNAs (lncRNAs) alterations; these factors and others provide susceptibility to develop multidrug resistance (MDR) [9]. The development of chemotherapeutic resistance is caused by MDR genes, including MDR1 and MDR2. MDR1 is also called ATP Binding Cassette Subfamily B Member 1 (ABCB1), encoded by P-glycoprotein (P-gp) that depends on Ca^2+^ efflux pump. It was linked to resistance against different chemotherapies such as actinomycin D, paclitaxel, anthracyclines, and vinca alkaloids [10].

Furthermore, resistance is associated with altered machineries including, autophagy and hypoxia reducing drug efficacy and naturally causing drug resistance [11]. S1P is another molecule that was correlated with cancer resistance, tumor development and underlying cellular transformation, apoptosis, metastasis, and angiogenesis of the tumor microenvironment [12]. S1P is synthesized from the phosphorylation of sphingosine in the presence of ATP as a source of γ-phosphate. S1P exhibits its role through either autocrine or paracrine pathway and mediates its action through five specific G protein-coupled receptors (S1PR1-5). It acts through binding to the S1PR1 receptor stimulating activation of cancer cell growth, tumorigenesis, angiogenesis, and metastasis [13].

Tumorigenesis is mediated by S1PR1 and was attributed to the promotion of downstream signal transducer and activator of transcription 3 (STAT3), interleukin-6, and NF-κB networks. Additionally, S1PR1-linked signaling activates other pathways such as PI3K/AKT, MAPK/ERK1/2, Rac, and PKC/Ca and decreases the expression of cyclic adenosine monophosphate (cAMP) [13]. Interestingly, tumor cells showed an elevated S1P in addition to its receptor S1PR1 which subsequently provokes drug resistance. Furthermore, the signaling of S1P via its receptor S1PR1 induces cancer cell survival by inducing anti-apoptotic pathways [14]. Thus, S1P and its receptor can be regarded as anti-cancer therapeutic targets to decrease cancer cell proliferation and, to aid in decreasing cancer cell resistance [15]. S1P is distributed in plasma, blood cells, as well as various cancer tissues [16]. The production of S1P is confined to two isoforms of kinase enzymes including SphK1 and SphK2 [17, 18]. Previous reports addressed elevated levels of sphingosine kinases in various cancer origins such as gastric, breast, pancreatic, and, lung carcinomas [13].

Therefore, targeting SphK1, and SphK2 by using inhibitors to produce low levels of S1P can be a novel protocol to minimize the cancer cell's resistance towards chemotherapeutic drugs. Both SphK1 and SphK2 share the same sphingosine binding site, but there are significant differences between them affecting the selectivity of the inhibitors [19, 20]. Many studies addressed novel selective inhibitors for SphK1 and SphK2. Nonetheless, many of developed inhibitors have off-target effects with lateral effects on other lipid or protein kinases. These sphingosine analogs pointed out low robustness and selectivity such as trimethyl-sphingosine (TMS), dimethyl-sphingosine (DMS), and Safingol [21]. TMS and DMS have selectivity for both SphK2 and ceramide kinase (CERK). DMS and TMS were suggested to be potential anticancer agents by controlling the cell growth-related signals, with significant impact when experimentally tested either in vitro or in vivo. Also, the present review discusses SphK1 gene expression among different carcinomas and associated impact on survival with elaborating the protein subcellular compartmentation, and protein–protein interactions. In addition, our team made an overview regarding S1P metabolism, functions, signaling, and transport. Furthermore, we demonstrated mechanisms of cancer resistance, and provided deep insight into S1P linked roles in cancer including different machineries of progression, metastasis, and cancer resistance.

We performed molecular docking of different inhibitors of SphK1 with revealing conclusive scoring for the best inhibitor with high inhibitory effect compared to control. These inhibitors attenuate the activity of SphK1 and subsequently decrease the production rate of S1P suppressing cancer cell resistance. Through our proposed preliminary pipeline and deep literature review we acquired, up to date all publicly available inhibitors along with their computational testing to address the most potential and reliable inhibitor that can be furtherly tested to ensure robust attenuation of S1P expression and enhancement the responsiveness of cancer towards chemotherapy.

## Bioinformatics framework of SPHK1

**Fig. 1:**
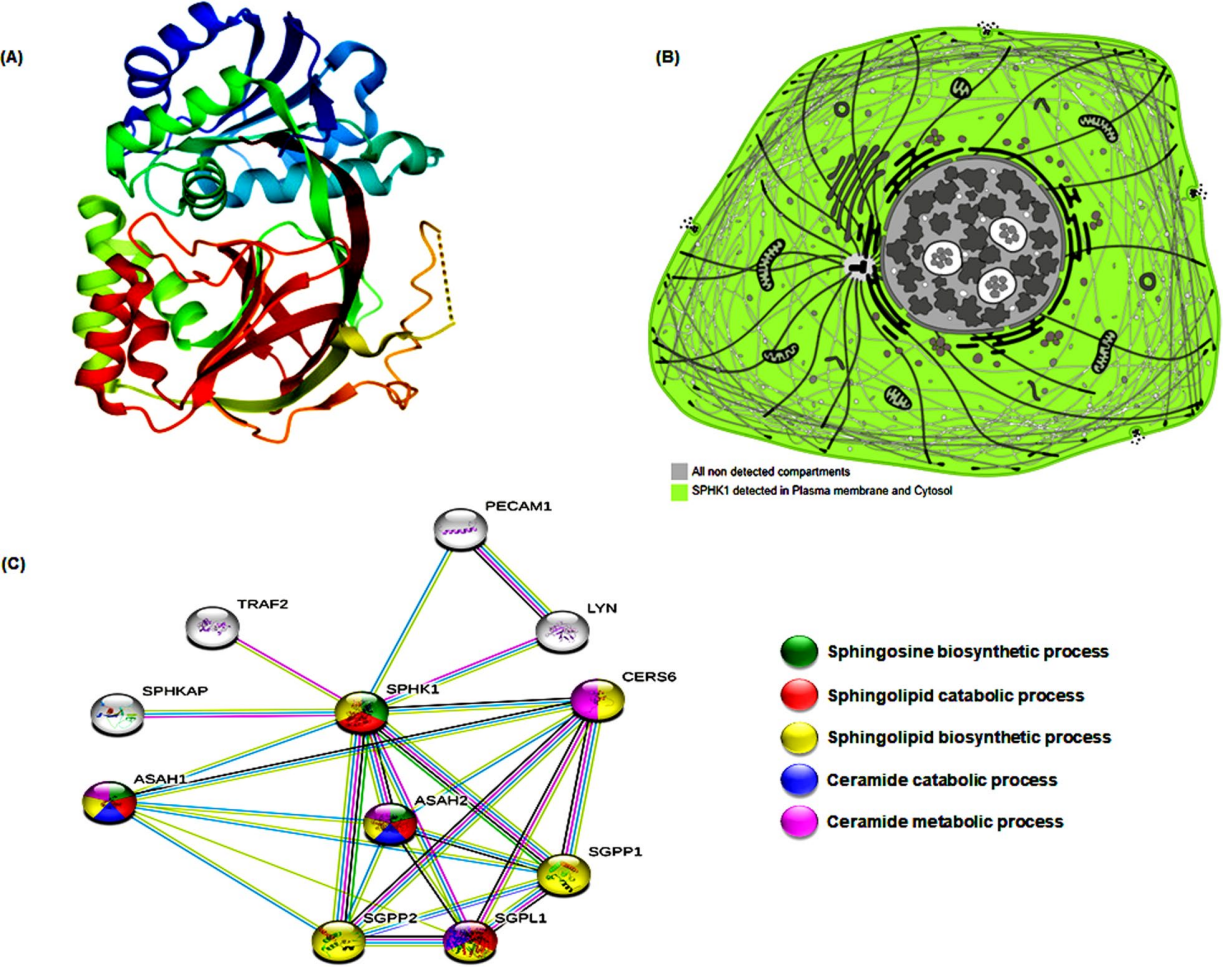
The bioinformatic framework analysis of SphK1. **A** The crystal structure of SphK1. **B** SphK1 subcellular localization in the cell, more abundance is relative to a darker color. **C** Protein–protein interactions of the SphK1 protein by STRING database. [Data source: UniProt database, The Human Protein Atlas, STRING version 11.0]

**Fig. 2:**
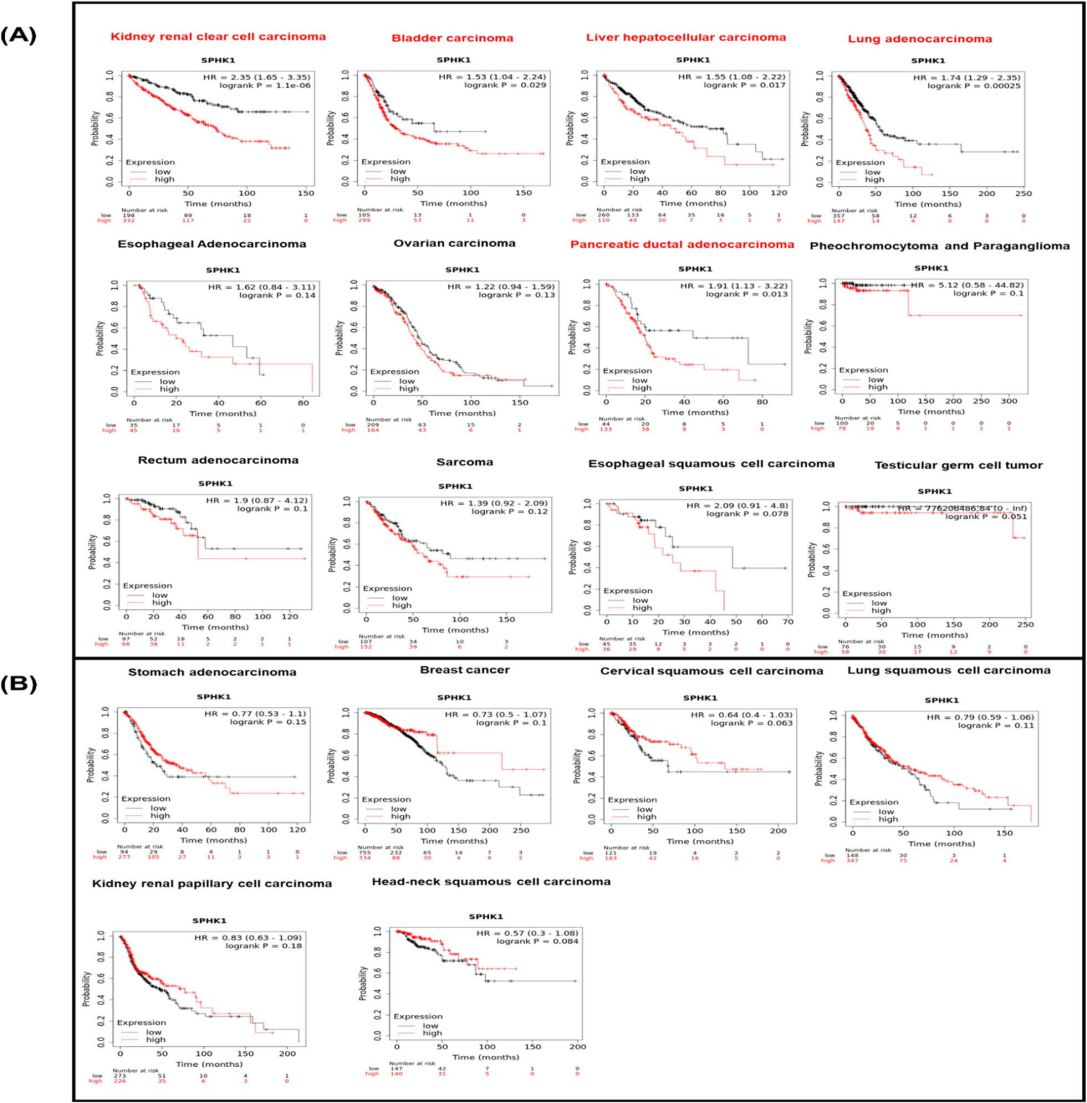
Kaplan–Meier survival plot using KM-plotter for SphK1 gene expression across different carcinomas, the upper partition (**A**) represents worse prognosis associated with low expression of the enzyme, while the lower partition (**B**) represents worse prognosis with high expression of the enzyme. Red-labeled cancers imply statistical significance in this type of cancer. [Data source: Kaplan‐Meier plotter database]

SphK1 gene [ENSG00000176170] has many aliases including (Sphingosine Kinase 1, SPHK, SPK 1, acetyl transferase SPHK1 and SK1). The gene includes 7 exons and is located within chromosome 17. SphK1 protein is comprised of 384 amino acids, with a molecular mass of 42,518 [UniProt Id: Q9NYA1], (Fig. 1A). The protein could be presented mainly in the cytosol as documented in the Human Protein Atlas, (Fig. 1B). Protein–protein network was performed using STRING database, (Fig. 1C). Furthermore, we could reveal the prognostic impact of SphK1 overproduction in the course of cancer through pan-cancer analysis using publicly accessible RNA-seq datasets from various human carcinomas. Accordingly, the Kaplan–Meier survival plot was conducted through KM-plotter (https://kmplot.com/analysis/), (Fig. 2) [22–24].

## Sphingosine-1-phosphate metabolism

Sphingolipids refer to the family of amphipathic lipids; the hydrophobic partition is attributed to ceramide. Ceramide is the precursor for the biogenesis of different sphingolipids, including S1P. S1P is considered a bioactive lipid that can be derived by ceramide deacylation through ceramidase, then undergo phosphorylated through sphingosine kinases named SphK1 and SphK2 that exist in cytosol and nucleus, respectively [25]. Ceramides can be synthesized within the endoplasmic reticulum through the de novo pathway. The process is dependent on serine palmitoyl transferase (SPT) that is able to condensate serine and palmitoyl-CoA forming 3-keto-dihydroshingosine [26].

**Fig. 3:**
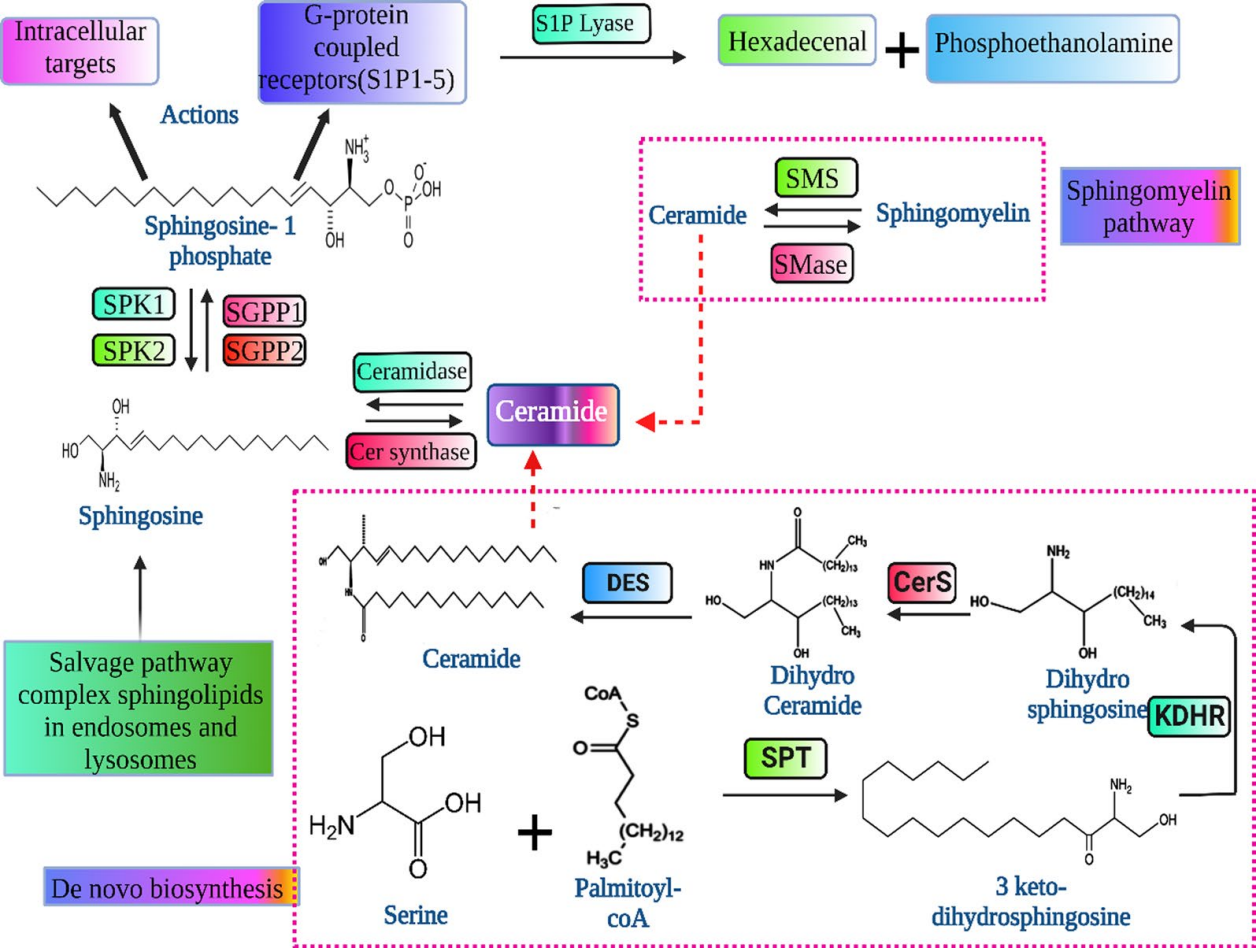
Overview of S1P metabolism. The de novo pathway begins with small molecules such as serine and palmitoyl-CoA and subsequently by the activity of SPT, KDHR, CerS, and dihydro-ceramide desaturase forming ceramide which can be utilized for S1P formation. The acidic environment of endosomes and lysosomes degradation of complex sphingolipids, including sphingomyelin, forms sphingosine, then are phosphorylated by SphK1 and SphK2. Furthermore, plasma membrane sphingomyelin by the action of sphingomyelinase to ceramide. SPT: Serine palmitoyl transferase; KDHR: 3-ketodihydrosphingosine reductase; CerS: ceramides synthases; SphK1/2: Sphingosine kinase 1/2; SGPP1/2: S1P phosphatase. The chemical structures used in the present illustration were drawn using ChemDraw Professional 21.0 software, and the figure was drawn by using Biorender https://www.biorender.com/

Then, by 3-ketodihydrosphingosine reductase (KDHR) reduction, di-hydrosphingosine is formed. After that, it is acetylated to form dihydroceramides by ceramide synthase (CerS). Eventually, ceramide is formed by dihydro-ceramide desaturase [27]. On the other hand, sphingomyelin content in the plasma membrane can release ceramides through the enzymatic activity of sphingomyelinase [27]. Furthermore, through the salvage pathway in the endosome and lysosome, complex sphingolipids, including sphingomyelin can be degraded to form ceramides that shuttled to the golgi apparatus to produce S1P [28], (Fig. 3).

In the context of SphK1 regulation, the enzyme is activated through extracellular signal-regulated kinase 1/2 (ERK-1/2) mediated phosphorylation signaling [29]. Also, while S1P is involved in NF-κB activation through PKCδ binding, it is reported that PKCδ could activate SphK1, forming S1P directly or indirectly by modulation ERK1/2 [30]. In normal conditions, S1P is depleted intracellularly to exert its effect on target proteins and then degraded irreversibly through S1P lyase (S1PL), forming hexadecenal and ethanolamine phosphate or reversibly dephosphorylated to sphingosine by the action of S1P phosphatase (SGPP1 and SGPP2) in addition to nonspecific lipid phosphate phosphatases. Sphingosine, in turn, can be utilized as a precursor to produce ceramide through ceramide synthase.

Interestingly, S1P showed a relatively higher concentration in blood and lymph compared to tissue [31]. Intracellular S1P action requires the export of ATP-binding cassette (ABC) C1, ABCG2, as well as spinster 2 (Spns2) to be functional. Additionally, S1P is exported generally due to active transport from erythrocytes through mfsd2B2. Another source of S1P is platelets that lack S1P lyase with the ability to release S1P via calcium- and ATP-dependent transporters. Furthermore, endothelial and lymphatic cells supply the blood with S1P through passive transport via the Spns2 transporter.

In addition, the amphipathic nature of S1P permits the molecule to be bound in the blood to carrier proteins such as high-density lipoprotein (HDL) and albumin with ratios of about 65% and 35%, respectively. Apolipoprotein M (ApoM) is considered the most common lipoprotein to which S1P has an affinity to bind, with observed decreased efficacy of S1P in ApoM-deficient HDL [32]. In the nucleus, S1P can be produced from ceramide with the aid of ceramidase and SphK2 [33]. S1P performs its roles according to the location of synthesis; for instance, intranuclear S1P produced through SphK2 mainly exerts an epigenetic effect [15].

## Functions of sphingosine-1-phosphate

S1P is a pluripotent lipophilic mediator; S1P is involved in numerous functions, including autoimmunity, inflammation, cardiovascular regulation, central nervous system (CNS), diabetes, cell cycle, and cancer [34, 35]. Multiple sclerosis is one of the autoimmune diseases where sphingosine-like molecule FTY720 can exert a favorable effect on relapsing and remitting the disease by attenuation of S1PR1 sequesters lymphocytes in lymph nodes, hindering their movement towards CNS and subsequent multiple sclerosis relapse [36]. Additionally, S1P has an association with macrophages' protection from apoptosis, modulates their trafficking, and enhances their anti-inflammatory capacity and apoptosis [37].

Furthermore, S1P promotes the recruitment of neutrophiles, eosinophils, mast cells, and monocytes. Moreover, S1P provokes the trafficking of dendritic cells and monocytes [38]. Also, S1P can modulate cystic fibrosis conditions [39]. In dendritic cells, the S1P axis is a downstream element in inflammation during sepsis mediated through protease-activated receptor 1 PAR1. Moreover, it was found that S1P can cause lethal septic shock [40]. Thus, it is not surprising that specific SphK1 inhibitors could enhance systemic inflammation and mortality associated with sepsis [41]. Similar to other lipids, S1P is associated with obesity and Type 2 diabetes (T2D) with different mechanisms according to diverse signaling types of S1P; this diversity made S1P capacity to affect insulin resistance in controversial ways [42].

S1P is one of the crucial players in the cardiovascular hemostasis process. Furthermore, many cardiovascular diseases have been linked to S1P, for instance, coronary artery disease and atherosclerosis. S1P is involved in myocardial infraction and heart failure [43]. S1P accounts for many impacts of HDL-bound S1P, such as anti-apoptosis, anti-inflammation, angiogenesis, nitric oxide (NO) production, and vasodilation [44]. Generally, cardiovascular disease prognosis can be linked to HDL content, to which S1P is bound, and can be considered as a biomarker for cardiac and vascular diseases with a significantly lower level of HDL in patients compared to healthy controls [45]. During embryogenesis, endothelial cells express mostly S1PR1 in an S1P-dependent manner. Lacking S1P1 during development causes critical defects in vascular morphogenesis that can be attributed to promoting the impact of S1P on endothelial proliferation, migration, angiogenesis, and vascular integrity along with apoptosis attenuation [46].

Furthermore, SphK2-derived S1P has a protective role against ischemic damage [47]. Nonetheless, heterozygous knockout of S1P lyase knockout hearts pointed out significantly improved functional recovery following ischemia/reperfusion [48]. The cardioprotective capacity of S1P was attributed to binding to the S1P3 receptor and enhanced nitric oxide (NO) production [49]. Additionally, S1P ameliorates atherosclerosis, which could be via NO production, attenuation of oxidative species, inflammatory chemokines formation, and their releases, such as TNFα, IL-6, IL-12, IFNγ, and MCP-1 [50]. Conversely, no obvious data in the context of S1P impact on the heart, while via S1P1, S1P can exert an impact on protein synthesis and cellular hypertrophy through MAPK and STAT3. Furthermore, S1P provokes vasoconstriction via interaction with vascular smooth muscle cells (VSMC) in mesenteric, cerebral, and coronary arteries while no impact on femoral and carotid arteries or aorta; this fluctuations can be attributed to the variation of expression of receptors and SphK1 [51].

S1P has a role in CNS through the protection of dopaminergic neurons as well as multifactorial roles in Parkinson’s disease and Alzheimer's disease (AD) [35]. S1P could modulate the survival and proliferation of various cells of the neural system including neurons and glial cells [52]. Additionally, S1P is involved in inflammatory reactions during neuroinflammation and is implemented in brain development [48]. S1P can attenuate the BACE1 enzyme, which is considered a significant player in the formation of Amyloid-β peptide (Aβ) and subsequent accumulation, which has severe consequences on brain health and linked AD [53]. Additionally, S1P produced in mitochondria via SphK2 was found to bind to PHB2 protein that is critical for the assembly of cytochrome-c oxidase as well as cellular respiration, while lack of S1P can lead to alterations in the respiratory chain and oxidative phosphorylation. Furthermore, S1P acts through receptor-mediated signaling through interaction with distinctly expressed G protein-coupled receptors (GPCR), named (S1PR1-S1PR5), which can provoke many effectors, such as MAPKs [54].

These receptors are most prevalent in cardiovascular machineries as well as immunity, while S1P4 has low expression in the lymph. S1P1 binds to the Gi/o alpha subunit of heterotrimeric G proteins. Conversely, S1PR2 and S1PR3 bind to Gi/o, Gq and G12/13. While S1P4 and S1P5 interact with Gi/o and G12/13 [55]. Signaling of S1P promotes phospholipase C as well as Ca^2+^ shuttle via Gq in addition to provoking Erks and PI3K. Furthermore, S1P attenuates adenylate cyclase through Gi [56]. It is responsible for the activation of Rho/actin cytoskeleton assembly via G12/13 [56].

HDL-bound S1P–S1PR1 entrapment in the plasma membrane decreases TNFα and underlying activation of NF-κB and ICAM-1 expression, in addition to the reduction of anti-inflammatory response through β-arrestin-mediated signaling [57]. S1P can promote protein kinase C delta (PKCδ) during the process of endotoxin-induced activation of NF-κB, which possesses various effects on inflammation and as an anti-apoptotic agent [56].

S1P can promote chemokines that correlate with angiogenesis and cytokines associated with proliferation and cell cycle regulators promoting cell survival [58]. Indirectly, S1P can inhibit histone deacetylases (HDACs), leading to elevation of NF-κB [59]. Also, S1P can promote the phosphatidylinositol 3-kinase PI3K/Akt pathway via its receptors, with subsequent effects favoring migration and angiogenesis and hindering apoptosis [60]. Additionally, S1P binds to S1PR1, forming a complex with platelet-derived growth factor receptor β (PDGFβ) that can promote cell migration through the ERK pathway [61].

Generally, S1P opposes the effect of ceramide. S1P is responsible for promoting proliferation, survival, cell growth arrest, cellular transformation, migration, epigenetic regulation, angiogenesis, and lymphangiogenesis [62]. S1P can mediate the trafficking of lymphocytes through activation of S1PR1, natural killer T cells aggression from lymph through S1PR5, and regulation of blood vessel permeability [63, 64]. S1P that is produced intranuclearly has an epigenetic role in histone acetylation as well as transcription modulation via exerting an inhibitory effect on HDAC1 and HDAC2, resulting in overexpression of cyclin-dependent kinase inhibitor p21 [65, 66]. Also, S1P attenuates the transcriptional regulator c-fos. Therefore, S1P can be considered one of epigenetic regulation machinery [53, 67, 68].

S1P regulates transcription factor peroxisome proliferator-activated receptor (PPAR)γ, which is involved in neovascularization [69]. Human telomerase reverse transcriptase (hTERT) is generally overexpressed in cancer; it is also responsible for telomere integrity. S1P formed by SphK2 can modulate telomer formation through stabilization of (hTERT), which subsequently increases cell survival [70]. S1P wasn’t considered a biological marker for cancer; this claim was attributed to its non-significant levels in plasma in several cancer samples. However, it was higher in ovarian carcinoma with a crucial role in lung cancer; in addition, it has a significant increase in early-stage prostate cancer detection [71, 72]. Additionally, S1P has an important role against ceramide to maintain cell homeostasis; ceramide is a tumor suppressor, apoptosis and autophagy promoting agent and inhibiting cell growth, whereas S1P is associated with anti-apoptotic, metastasis,–mesenchymal transition (EMT), angiogenesis and chemotherapy resistance [73, 74].

Thus, fluctuations in balance among S1P, ceramide, and their enzymes are associated with cancer prognosis and angiogenesis, particularly with a metabolic shift toward S1P production during carcinogenesis. S1P is also responsible for providing the cancerous microenvironment [75]. Therefore, many chemotherapeutic drugs were designed to promote ceramide production. In our study, we will discuss the potential inhibitory effect of several SphK1 suppressors as an adjuvant treatment against cancer to tackle chemoresistance.

S1P was found to be increased in the MCF-7 breast cancer cell line and was associated with apoptosis inhibition and promoting growth via serum response element that interacts with the c-fos gene that initiates invasive growth in cancer [62, 76]. Moreover, S1P enhances insulin-like growth factor (IGF2) production and activity; this alteration was linked to an increased susceptibility of many carcinomas through IGF2-mediated promoting insulin growth factor 1 receptor (IGF1R) or hybrid receptors that may promote tumorigenesis. Moreover, IGF2 through the MAPK pathway can promote IGF1R, causing activation of genes associated with growth and rapid proliferation. SphK1 enzyme is up-regulated in breast cancer with subsequent elevation of S1P and oncogenic phenotype with Ras-dependent transformation of tumor cells [77].

Additionally, S1P induces the proliferation and migration of hematopoietic stem cells (HSCs) [77, 78]. Many of the multifactorial roles of S1P are linked to microenvironmental niche modulation. However, the specific receptors of S1P and subsequent events in a niche can be varied depending on cancer types for instance, in bone metastasis breast cancer, S1PR1 and IL-22R1 are up-regulated [62]. Neovascularization by the VEGF-A-VEGFR2 pathway can also be stimulated by S1PR1 activation [79]. The progression of cancer by S1P can be related to the regulation of HDAC1/2 enzymes and subsequent modulation of gene transcription to promote cancer.

## Sphingosine-1-phosphate signaling

S1P is a bioactive pleiotropic sphingolipid mediator, also called glycosphingolipids. The signaling pathway of this mediator is considered a survival key for the cell to stay alive, proliferate, and migrate [7]. S1P is mainly produced from ceramide hydrolysis by ceramidase enzyme followed by the action of SphK1 to produce S1P. S1P can exert its action by intracellular targets or extracellularly through 5 G-protein coupled receptors (S1PR1-5) [80].

S1P receptors have distinctive G-protein-coupling activities that include five subtypes: S1PR1, S1PR2, S1PR3, S1PR4, and S1PR5 [81]. S1PR1 combines completely with the G_i/o_ family, while S1PR2 couples to G_i/o_, G_12/13_ in addition to the G_q_ family. Generally, S1P was linked to various cellular processes such as control of cell division, neo-vascularization, and migration. Also, S1P is involved in cytoskeleton assembly, trafficking of immune cells, and mitogenesis. Furthermore, S1P receptors are involved in immunological regulation, such as attenuation of T cells' innate immune responses. Nevertheless, S1P receptors are expressed in a large spectrum of tissues, and each tissue subtype has a unique cell specialization; for instance, S1PR1 in lymphocytes, S1PR5 is mostly found in the spleen and the central nervous system (CNS) especially in white matter, whereas S1PR4 is restricted to lymphoid and hematological organs [68].

## Sphingosine-1-phosphate transport

S1P uses two machineries to exhibit its physiological responses: (1) it is exported out of the cell and transported to exert a paracrine (or autocrine) effect, or (2) it can bind to intracellular targets and exert a response. S1P concentration gradients in the body might result from the local production and export rate of this bioactive sphingolipid [82]. It is crucial to determine the chemical composition of these transporters, given the potential role they may play in creating such gradients. A report indicates that Spinster 2 (SPNS2) and ATP-binding cassette (ABC) transporters are involved in transporting S1P proteins [83]. The ABC transporter family is a significant collection of membrane-embedded proteins that are involved in the transport of a wide spectrum of molecules, including lipids and cytokines, including S1P. The includes ABCA1 and ABCG2 along with ABCC1.

Lipids or lipid-related molecules transport, such as cholesterol as well as phospholipids transport, were attributed to approximately 50% of ABC [84]. S1P is a charged molecule that cannot diffuse across membranes and is carried through active or passive transport machinery to exhibit its effects [85]. The usefulness of ABCC1 in S1P transport was established in human as well as rodent mast cells, making it a pharmacological candidate to be evaluated through its inhibitor MK571, which substantially attenuated S1P release [86]. Release of S1P from mast cells is conducted via constitutive and stimulated release in an ABCC1-dependent manner; stimulated release is initiated by antigen stimulation [16, 41]. Estradiol was found to induce S1P release from MCF7 cells; this action is attenuated through pharmacological suppression of ABCC1 or ABCG2 genes. Released S1P is associated with cancer development as well as multidrug resistance [64].

Various machineries participate in S1P accumulation in blood in associated form with HDL particles such as retinoic acid/cAMP [87]. This accumulation can be attenuated through the administration of ABCA1 siRNA and non-selective ABCA1 inhibitor glibenclamide. Additionally, MK571 and glibenclamide both act on human vascular endothelial cells, hindering S1P release. Despite the fact that ABC transporters were once thought to act as pore-forming proteins with a hydrophilic pore that works as a vehicle to transport hydrophilic substrates across the membrane, it is now believed that they act as flippases that transport lipid-soluble compounds from the inner to the outer plasma membrane [88]. They are now considered to be a crucial link in S1P export signaling [89].

ABC proteins machinery for various lipid molecules transport including S1P, are ideal for the export of lipids by coupling this activity with a possible cargo action for substrates onto acceptors**.** Firstly, the location of S1P in the outer leaflet following flopping is potentially consistent with the lipid's ability to perform an autocrine role by attaching to an S1PR [90]. Second, S1P needs to be discharged into an aqueous media to perform paracrine signaling tasks. ABC transporters are thought to carry out this activity by transferring the substrate (S1P) from the inner leaflet to an acceptor molecule like albumin or APO (in HDL). Third, S1P might be loaded onto an extracellular acceptor after being removed from the outer leaflet [91].

On the other hand, SPNS2 participates in S1P transport as one of the main facilitator superfamilies (MFS) of transporters [41]. Evidence was based on the projected amino acid sequence. Given that it resembles the characterized V-shaped ABC transporters with included 12 transmembrane helices, the structure of MFS may be suitable for such a function [92].

## Role of sphingosine-1-phosphate on cancer cell progression and resistance

Resistance could be categorized into primary and secondary; primary arises from the tumor cells before therapy exposure, while secondary is attributed to tumor adaptation to the treatment, for instance, elevated expression of target proteins [93]. Cancer drug resistance is commonly attributed to genomic alterations. On the other hand, types of resistance machinery include EMT, signaling pathway bypass, drug efflux activation, drug entry impairment and upregulating the levels of S1P [2, 3, 6, 94]. S1P has a significant effect in increasing carcinogenesis, invasion, migration, survival, and metastasis [95]. S1P induces cancer cell resistance towards chemotherapeutic drugs. Cancer cells show increased S1P levels as well as a decrease in the expression of sphingosine and ceramide due to their pro-apoptotic characteristics [96].

The levels of S1P in normal cells are sustained via a balance between either activation of S1P lyase, which degrades S1P irreversibly, or sphingosine kinase (SphK1 & SphK2) activation. Whereas cancer cells induce high expression of S1P and its SphK1/2 enzymes to increase growth, and angiogenesis [97]. Furthermore, several studies showed that S1P stimulates the activity of p-glycoprotein through activation of Abcb1 transport by S1PR1 and S1PR3 receptors at the brain cancer cell surface (RBE4) [95]. Also, SphK1 overexpression upregulates the expression levels of Abcb1 and Abcb1b mRNA that are translated into P-gp [98].

P-gp is a multi-drug resistance protein that causes the efflux of chemotherapy from the interior of cancer cells to the exterior. On the other hand, stimulating S1P signaling and its production in interstitial fluids such as lymphatic fluid induces metastasis [98]. Furthermore, in breast tumor cells, high S1P concentrations have an impact on lymph node metastasis [76]. Consistently, patients with ER^+^ breast cancer who posse high expression levels of SPHK1 exhibit lower survival rates and increased chemoresistance [62].

**Fig. 4:**
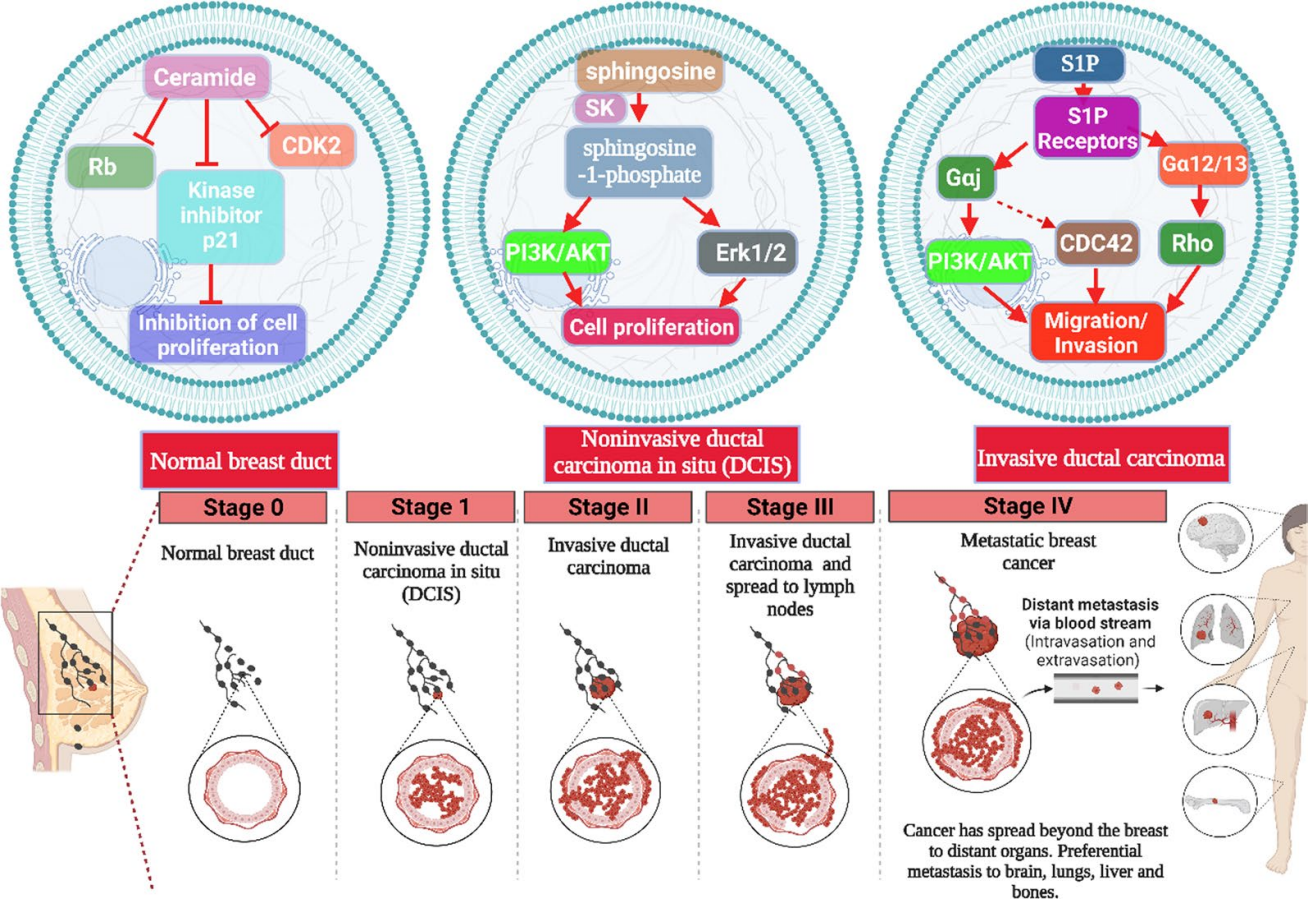
Effect of S1P on cancer cell proliferation. The figure was drawn by using biorender https://www.biorender.com/

**Fig. 5:**
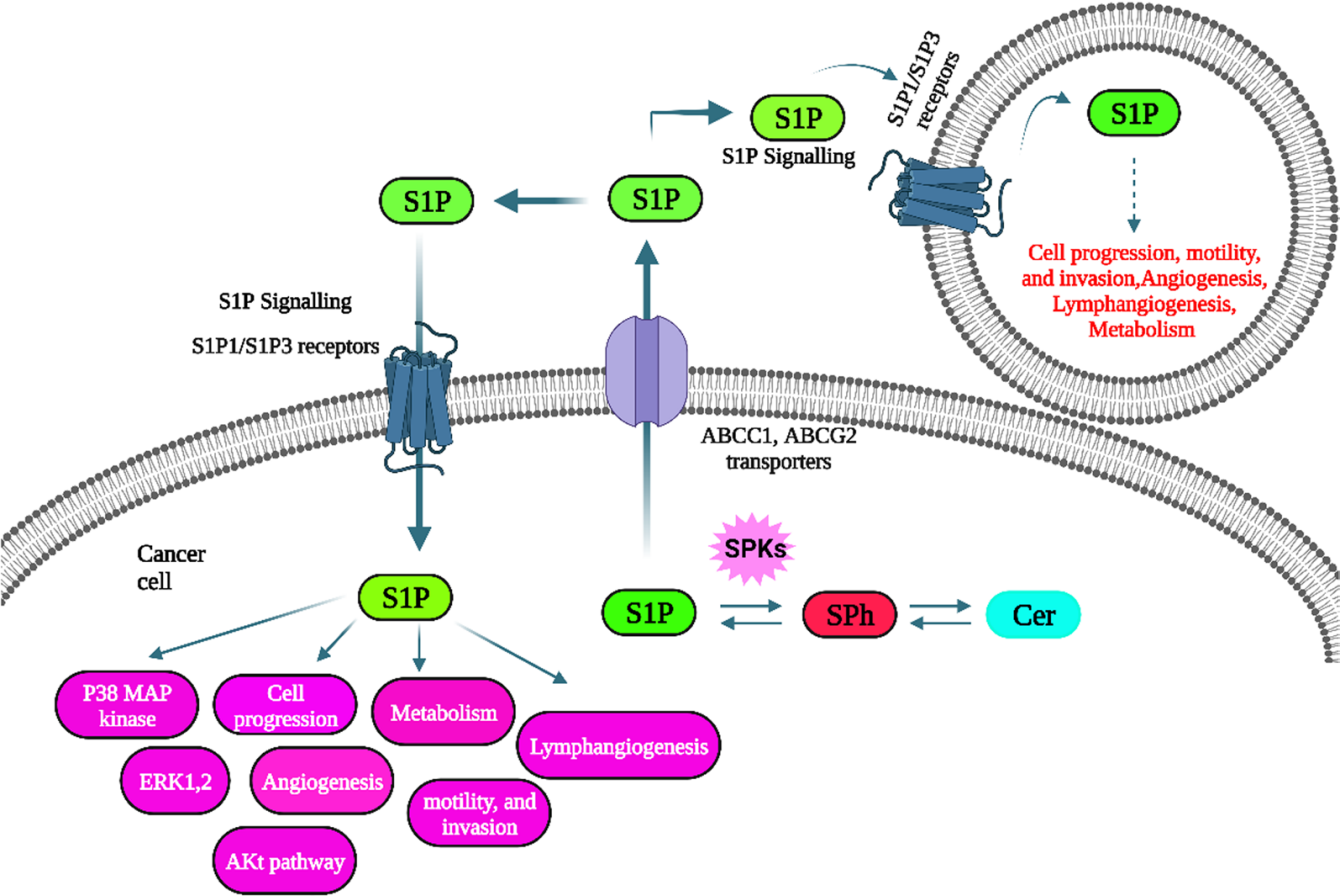
S1P involvement in drug resistance. The figure was drawn by using biorender https://www.biorender.com/

S1P induces the activation of extracellular signal-regulated kinase 1/2 (ERK1/2) enzymes, which subsequently activate the S1P receptor (S1PR) on the surface of cancer cells, leading to enhanced cancer cell proliferation, (Fig. 4), [76]. Extracellular S1P activates G protein-coupled receptors that trigger activation of survival or antiapoptotic signaling pathways, including protein kinase B (Akt)/mechanistic target of rapamycin (mTOR), CDC42 GTPases, and MAPK pathways [13, 99]. Interestingly, S1PR1 and S1PR2 pointed out high expression levels in patients with glioblastoma multiforme (GBM) [100]. S1P induces pancreatic cell proliferation and migration via c-Src pathway activation [101]. High concentrations of S1PR1/S1PR3 and ERK1/2 cause chemoresistance of breast tumor cells towards tamoxifen (Fig. 5) [76, 102]. The binding of S1P with S1PR3 promotes the phosphorylation of ERK1/2 and encourages its localization via an S1P3/ p21-activated protein kinase 1(PAK1)-dependent pathway in breast cancer cell models.

The high expression level of SphK1 induces cancer cell movement, migration, and invasion by controlling the movement of actin from focal adhesions to membrane ruffles and lamellipodia, which is necessary for migration [103]. Through S1P, actin is redistributed into membrane ruffles and encouraged to migrate, promoting MCF-7 cell migration [104].

Interestingly, the SphK1 siRNA inhibitor decreases the expression of S1PR3 in MCF-7 cells and prevents the development of the migratory phenotype [105]. In conclusion, the literature suggests that S1P inhibition or down-regulated SphK1 expression can be a promising approach to decrease cancer cell resistance.

## S1P as a therapeutic target for chemoresistance treatment

Cancer cells release S1P as a signaling molecule to regulate and modify the cellular functions of tumor microenvironment phases, including initiation, progression, growth, invasion of the tumor, and cellular communications [106]. S1P signaling augments cancer cell proliferation, chemoresistance, and metastasis [107, 108]. The elevation in S1P level was obtained in several cancers, including ovarian, prostate, colorectal, breast, and HCC [21, 109, 110]. HER2-positive breast cancer cells are characterized by high production of 17β-estradiol (E2) [111].

Interestingly, the relation between S1P production and E2 is very strong; 17β-estradiol (E2) binds to estrogen receptor (ER), enhancing the production of S1P through ABCC1 and ABCG2 transporters [112]. Then, S1P binds to its receptors and enhances the ERK1/2 pathway, which downregulates several machineries, including apoptosis and autophagy, promoting breast cancer cell growth, progression, and metastasis [111]. However, patients with breast cancer type ER-negative have higher concentrations of the enzyme SphK1, which is strongly associated with a high cancer proliferation rate and poor prognosis [21]. Conversely, breast cancer cells with ER-negative (MDAMB- 453) express high levels of Human epidermal receptor-2 (HER2), which facilitates S1PR4 stimulation to the ERK1/2 pathway. Accordingly, the downregulation of apoptosis and autophagy process and enhancement of cancer cell proliferation and survival [62].

ER-negative breast cancer and skin cutaneous melanoma survival and progression are linked with high expression levels of S1PR4 and SphK1 [113]. Therefore, the signaling pathway S1P/SphK1 can be an important target for therapeutic intervention in the context of cancer cell resistance [114]. SphK1 inhibitors include different types such as 1ipid-based, amidine-based, pyrrolidine-base, and natural source. Among those compounds, PF-543 was suggested as a potent SphK1 suppressor with a K_i_ of 3.6 nM. It decreases the growth, survival, and resistance of MDA-MB-231 triple-negative breast cancer cells and LM2-4 cells by inhibition of AKT pathway, p38 MAP kinase pathways, and ERK pathway. So, PF-543 is considered a potent anti-cancer drug and reduces the chemoresistance of cancer cells [105]. Other agents tackle the production of S1P and its signaling, such as FTY720. It is a synthetic sphingosine analogue generated from a chemical modification of a natural product [myriocin (ISP-I)] that is obtained through the cultivation of a fungal broth culture *Isaria sinclairii* [115].

It is used as a chemotherapeutic agent against cancer. Another example of SphK1 inhibition, SK1-I, is used as an anticancer by reducing cancer cell S1P levels, inducing cancer cell apoptosis through activation of caspases-3 and caspases-9, and reducing both hem-angiogenesis and lymph-angiogenesis [116]. SK1-I acts as a chemo-sensitizing agent via decreasing ERK1/2 and Akt pro-survival signaling [117]. Also, SK1-I represses the proliferation of colon cancer and breast cancer cells through upregulation of TP53 tumor suppressor protein and pro-apoptotic, triggering autophagy and cancer cell death. Owing to its high solubility and potent cytotoxic effect, SK1-I was applied in vivo in animal disease models [117]. Up to date, SphK1 inhibitors are depicted in Table 1 and will be overviewed and checked for their activity as an anticancer drug using molecular docking.

**Table 1:** Docking energy scores and amino acids involved in the binding site for SphK inhibitors with the active site of SphK1

Name of inhibitor	IUPAC name	Docking score (kcal/mol)	Chemical Structure	Amino acids involved in binding
*Lipid-like small molecules SphK inhibitors*				
Sphingoguanidine-based SphK				
LCL351	*N*-[(4E)-1-hydroxyoctadec-4-en-2-yl] guanidine	− 7.7789		THR 54
SLR080811	(2S)-2-[3-(4-octylphenyl)-1,2,4-oxadiazol-5-yl] pyrrolidine-1-carboximidamide; hydrochloride	–		–
SLM6031434	(2S)-2- [3- [4- (Octyloxy)-3-(trifluoromethyl) phenyl]-1,2,4-oxadiazol-5-yl]-1-pyrrolidinecarboximidamide hydrochloride	–		–
SLC5111312	(2S,3S)-3-hydroxy-2-(3-(6- (pentyloxy) naphthalen-2-yl)-1,2,4-oxadiazol-5-yl) pyrrolidine-1-carboximidamide hydrochloride	–		–
SLP120701	(S)-2-(3-(4-octylphenyl)-1,2,4-oxadiazol-5-yl) azetidine-1-carboximidamide hydrochloride	–		–
SLC4011540	2-(3-[4-({4-[4- (difluoro methyl) phenyl]-1,3-thiazol-2-ylamino)phenyl]-1,2,4-oxadiazol-5-yl methyl)pyrrolidine-1-carboximidamide	− 9.3994		ARG 191 GLY 342
SLP7111228	(2*S*)-2-[[3-(4-octylphenyl)-1,2,4-oxadiazol-5-yl] methyl] pyrrolidine-1-carboximidamide; hydrochloride	–		–
Amidine-based SphK inhibitors				
VPC96091	(2*S*-1-(4-dodecylbenzoyl) pyrrolidine-2-carboximidamide	–		–
VPC94075	*N*-[(2S)-1-amino-1-iminopropan-2-yl]-4-octylbenzamide; hydrochloride	–		–
Compound 28	1-carbamimidoyl-*N*-(4-dodecylphenyl) cyclopropane-1-carboxamide	− 8.2461		N 44- ASP 178 & N 56- ASP 178 & ARG 191
Compound 1a	1-(4-dodecylbenzoyl) pyrrolidine-2-carboximidamide	− 8.0355		ASP 178 SER 168 ARG 191
Piperidine-based SphK inhibitors				
Compound 82	(1–2-[4-({2-[4-(trifluoromethyl) phenyl]-1,3-thiazol-5-yl amino)phenyl]ethyl piperidin-2-yl)methanol	− 7.6419		GLY 113 ARG 185
RB-005	1-[2-(4-octylphenyl)ethyl] piperidin-4-amine	− 7.3644		GLY 113
SK1-5c	2, 2-dimethyl-4S-(1-oxo-2 hexadecyn-1-yl)-1, 1-dimethylethyl ester-3-oxazolidinecarboxylic acid	− 8.9416		GLU 343
Pyrrolidine-based SphK inhibitors				
CHJ01	(2R,3S,4S)-4-amino-2-tetradecylpyrrolidin-3-ol; dihydrochloride	–		–
Compound 51 (SK1-IN-1)	(2S, 3S)-N-(S)-1-(4- (5-(2-cyclopentylethyl)-1, 2, 4-oxadiazol-3-yl) phenyl) ethyl) hydroxypyrroli-dine-2-carboxamide	− 8.0534		GLY 26 GLY 111 SER 112
PF-543 Reference molecule	[(2R)-1-[[4-[[3-(benzenesulfonylmethyl)-5-methylphenoxy] methyl] phenyl]methyl]pyrrolidin-2-yl]methanol	− 8.9563		GLY 26 SER 112 LYS 27 ARG 191 GLY 342
Naphthalene based SphK inhibitors				
SLC5091592	(2*S*)-2-[3-[6-[[3-(trifluoromethyl) phenyl]methoxy]naphthalen-2-yl]-1,2,4-oxadiazol-5-yl]pyrrolidine-1-carboximidamide;hydrochloride	–		–
Amino alcohol-based SphK inhibitors				
SK1-I	(2R, 3S, 4E)-*N*-methyl-5-(4-pentylphenyl)-2-aminopent-4-ene-1, 3-diol)	− 6.7484		ARG 191 GLY 342
DHS (Safingol)	[(2*S*,3*S*)-1,3-dihydroxyoctadecan-2-yl] azanium	− 6.9007		ASP 81 ASP 178 ARG 191 ASP 178
DMS	(*E*,2*S*,3*R*)-2-(dimethylamino) octadec-4-ene-1,3-diol	− 7.0454		GLU 182 ARG 191
K145	3-(2-amino-ethyl)-5-[3-(4-butoxyl-phenyl)-propylidene]-thiazolidine-2,4dione	− 6.7742		GLU 343 GLU 343
SG12	2-amino-4-(4-octylphenyl) butane-1,3-diol	− 6.6408		GLU 343 GLU 182
SG14	*N*-[2-hydroxy-1-phenyl-5-(pyrrolidin-1-yl) pentan-3-yl] octadecanamide	− 11.4135		GLY 113
Amgen 82	2-(hydroxymethyl)-1-2-[4-({4-[4-(trifluoromethyl) phenyl]-1,3-thiazol-2-yl amino) phenyl] ethyl piperidin-4-ol; trifluoromethane	− 8.2950		GLY-133 & ASP 178& GLY 82 & ILE 174
FTY720	(2-amino-2-[2-(4-octylphenyl) ethyl] propane-1, 3-diol)	− 7.1602		LEU 268 ARG 191 GLY 342
(S)-FTY720 vinyl phosphonate	[(3S)-3-amino-3-(hydroxymethyl)-5-(4-octylphenyl) pent-1-enyl]phosphonic acid	− 7.0428		GLY-111 & SER-112
ROMe (R)-FTY720-OMe)	(2R)-2-amino-2-(methoxymethyl)-4-(4-octylphenyl) butan-1-ol	− 6.8914		SER 79 GLY 82
*Non-lipid like small molecule SphK inhibitors* Benzene sulfonamide-based SphKs inhibitors				
MP-A08	4-Methyl-*N*-[2-[2-[(4-methylphenyl) sulfonyl] amino] phenyl] amino] methyl]phenyl] benzene sulfonamide	− 7.2889		GLU 182 GLY 82
SKI-II	4-[[4-(4-chlorophenyl)-1,3-thiazol-2-yl] amino]phenol	− 6.5171		ASP 178 MET 272
11b	6-(hydroxymethyl)-3-[(1E)-3-(4-[4-(naphthalen-2-yl) pyrimidin-2-yl] amino- phenyl)-3-oxoprop-1-en-1-yl]-1,2-dihydroquinolin-2-one	− 8.2255		SER 112
SKI-I	*N*-[(*E*)-(2-hydroxynaphthalen-1-yl) methylidene amino]-3-naphthalen-2-yl-1*H*-pyrazole-5-carboxamide	− 8.2044		SER 168
SKI-I-Asp	3-[(E)-([3-(naphthalen-2-yl)-1H-pyrazol-5-yl] formamide-amino)methyl]naphthalen-2-yl 2-(methoxy methoxy)benzoate	− 8.1246		GLY 82 ARG 191 GLY 25
SKI-178	*N*-[(*E*)-1-(3,4-dimethoxyphenyl) ethylidene amino]-3-(4-methoxyphenyl)-1*H*-pyrazole-5-carboxamide	− 8.8007		MET 272
SK-F	*N*-(4-octylphenyl) benzamide	− 7.9155		ILE 174
Opaganib ABC294640	3-(4-chlorophenyl)-*N*-(pyridin-4-ylmethyl) adamantane-1-carboxamide	− 6.6796		GLY 25
ABC294735	3-(4-chlorophenyl)-*N*-[(3,4-dihydroxyphenyl) methyl] adamantane-1-carboxamide	− 7.3269		ASP-178
CB5468139	*N*-(3-chloro-1,4-dioxonaphthalen-2-yl)-*N*-cyclohexyl acetamide	− 6.0431		GLU 343
ST-1803	4-methyl-*N*-[4-(1,3-thiazol-2-yl)-1,3-thiazol-2-yl]-1,3-thiazol-2-amine	− 5.8147		ILE 174 ILE 174
*SphK inhibitors from natural sources*				
Pachastrissamine (jaspine B)	(2S,3S,4S)-4-amino-2-tetradecyloxolan-3-ol	− 7.6449		ARG 56 GLU 55 ALA 60
F-12509a	(6aR,12aR,12bS)-10-hydroxy-4,4,6a,12b-tetramethyl-1,2,3,4a,5,6,12,12a-octahydrobenzo[a]xanthene-8,11-dione	− 5.9055		ARG 191 ARG 191
B-5354C	[(Z)-tetradec-7-enyl] 4-amino-3-hydroxybenzoate	− 8.2508		GLY 82 & PHE 192
Balanocarpol	(1*R*,8*S*,9*S*,16*R*)-8,16-bis(4-hydroxyphenyl)-15-oxatetracyclo [8.6.1.0^2,7^.0^14,17^] heptadeca-2(7),3,5,10(17),11,13-hexaene-4,6,9,12-tetrol	− 5.7554		ARG 57 & GLU 55 & ALA 110
Icaritin	3,5,7-trihydroxy-2-(4-methoxyphenyl)-8-(3-methylbut-2-enyl) chromen-4-one	− 6.2876		GLU 343 SER 79 ARG 185
Hispidulin	5,7-dihydroxy-2-(4-hydroxyphenyl)-6-methoxychromen-4-one	− 6.3303		MET 272
Peretinoin	(2*E*,4*E*,6*E*,10*E*)-3,7,11,15-tetramethylhexadeca-2,4,6,10,14-pentaenoic acid	− 6.6058		ARG 57
Pristimerin	methyl (2*R*,4*aS*,6*aR*,6*aS*,14*aS*,14*bR*)-10-hydroxy-2,4*a*,6*a*,6*a*,9,14*a*-hexamethyl-11-oxo-1,3,4,5,6,13,14,14*b*-octahydropicene-2-carboxylate	− 7.1293		GLU 182 ARG 191 ARG 191
Suramin	8-[[4-methyl-3-[[3-[[3-[[2-methyl-5-[(4,6,8-trisulfonaphthalen-1-yl) carbamoyl] phenyl] carbamoyl] phenyl] carbamoyl amino] benzoyl] amino] benzoyl] amino]naphthalene-1,3,5-trisulfonic acid	− 7.0881		ASP 81 MET 272 ARG 24 ARG 185 ARG 24
Ellagic acid (EA)	6,7,13,14-tetrahydroxy-2,9-dioxatetracyclo [6.6.2.0^4,16^.0^11,15^] hexadeca-1(15),4,6,8(16),11,13-hexaene-3,10-dione	− 4.9543		SER 79 LEU 83 GLY 113 (A) GLY 113 (A
Epigallocatechin-3-gallate (EGCG)	[(2*R*,3*R*)-5,7-dihydroxy-2-(3,4,5-trihydroxyphenyl)-3,4-dihydro-2*H*-chromen-3-yl] 3,4,5-trihydroxybenzoate	− 6.9770		GLY 113 GLY 342 GLU 343

The chemical structures in the table were drawn using ChemDraw Professional 21.0 software

## Types of potent anti-cancer compounds that are based on inhibition of sphingolipid

SphKs play an important role in many diseases, so they were recognized as a promising therapeutic target. The sphingosine binding site is the same in SphK1 and SphK2, nonetheless, there are significant differences between them affecting the selectivity of the inhibitors [19, 20]. Many studies designed new selective inhibitors for SphK1 and SphK2, however, many of them have off-target effects on other lipids or protein kinases. SphK1 inhibitors were developed early in the nineteens (Sphingosine Analogs) that posse low robustness and specificity, such as trimethyl-sphingosine (TMS), dimethyl-sphingosine (DMS), and Safingol [117].

TMS and DMS have selectivity for both SphK2 and ceramide kinase (CERK). DMS and TMS were suggested to be potential anticancer agents by controlling the cell growth-related signals with significant inhibitory effects on tumors either in vitro or in vivo. Safingol affects other proteins, such as the protein kinase C (PKC) and ceramide synthase (CerS). On the other side, SphKs inhibitors were used as antiviral compounds [118]. Later, different inhibitors were developed, and the most promising inhibitors will be classified according to the structure, as shown in Table 1, and discussed in the upcoming sections according to their selectivity.

## Lipid-like small molecules SphK inhibitors

### Sphingoguanidine-based SphK inhibitors

Inhibitors that contain a base of sphingoguanidine as a polar moiety along with a sphingolipid backbone include LCL351, SLR080811, SLP120701, and SLM6031434, Table 1. It was believed that guanidine could interact with ATP directly in some enzymes' catalytic centers and prevent the phosphorylation reaction [119].

### LCL351

LCL351, L-erythro-2-N-(1’-carboxamidine), is the most effective agent over all the category compounds. It exhibited half-maximal inhibitory concentration (IC_50_) of 40 and 300 nM towards both SphK1 and SphK2, respectively. There are some modifications exerted on sphingosine to generate the LCL351 molecule upon the sphingosine hydrophilic head with the use of amine-guanidine and the subsequent alteration of its stereochemistry [119]. Studies of the structure–activity correlation demonstrated that the removal of the pyrrolidine hydroxyl group in sphingoguanidine-based inhibitors plays a role as a molecular guide to target SphK2 inhibition in more potency as compared to SphK1 [120].

Additionally, added methylene between the oxadiazole and pyrrolidine rings acts as a spacer and target inhibitor more towards SphK1 [19]. LCL351 triggers SphK1 degradation and reduces plasma S1P concentration as well as increases ceramide species levels along with pro-inflammatory cytokine elevation and alleviating infiltration of neutrophils [121]. However, using inhibitory concentration in vitro has no significant effect on apoptosis and cell cycle. It is observed that LCL351 can not only decrease the S1P levels in mice tissues with a long life time, but also, it has an impact on protecting tissues from inflammation [119].

### SLR080811

SLR080811, (S)-2-(3-(4-octylphenyl)-1,2,4-oxadiazol-5-yl) pyrrolidine-1-carboximidamide), was identified by modifying the structure of VPC96091, amidine moiety was replaced with guanidine isostere [122]. It is selective sphingosine competitive of SphK2 with a Ki value of 1.3 μM and is fivefold selective for SphK2 [123]. SLR080811 showed no selectivity for CERK or DAGKα, but no further testing was done for other enzymes. SLR080811 reduces the levels of S1P in both wild-type and SphK1 null cells but not in SphK2 null cells [117].

In ovarian cancer cell lines, including U937 and SKOV3, SLR080811 pointed out increased sphingosine, di-hydro sphingosine, and C16 ceramide. Additionally, two analogs of SLR080811 were designed, SLM6031434 and SLC5111312, using a docking program to generate SphK inhibitors utilizing SLR080811 as a template [117]. There are some modifications to the structure of SLR080811 to generate SLM6031434, including the incorporation of 39-trifluoromethyl moiety on the phenyl ring and an ether bond to the 49-octyl group on its structure [124]. Interestingly, SLM6031434 pointed out more potency than template SLR080811 [124]. The assembly of a 3-OH on the pyrrolidine ring, in addition, the 4-octyl phenyl moiety is replaced with a 6-pentoxylnaphthy of SLR080811 generating SLC5111312. SLM6031434 and SLC5111312 are found to have more selection characteristics toward SphK2, pointing out increased S1P concentrations in mice serum with K_i_ 0.4 μM and 1 μM, respectively [124].

### SLP120701

SLP120701, (S)-2-(3-(4-octylphenyl)-1,2,4-oxadiazol-5-yl) azetidine-1-carboximidamide hydrochloride), was considered as a selective inhibitor of SphK2 (K_i_ = 1.2 μM). There are some modifications to the SLR080811 template to produce the SLP120701, including replacing pyrrolidine with azetidine ring (smaller four-membered ring) [125]. It pointed out an ability to decrease the levels of S1P and sphingosine in U937 cells. Whereas, In vivo, it exhibited a half-life time of 8 h and increased the circulating S1P in mice. Also, SLP120701 has an anti-proliferative activity against breast cancer [126].

### SLC4011540

SLC4011540, (S)-(2-((3-(4-((4-([1,1′-Biphenyl]-4-yl)thiazol-2-yl)amino)phenyl)-1,2,4-oxadiazol-5-yl)methyl) pyrrolidin-1-yl) (amino) methaniminium is considered as guanidine compounds containing aminothiazole with the capability to inhibit both SphKs with K_i_ of 120 nM and 90 nM for SphK1 and SphK2, respectively [127].

SLC4011540 skeleton is an oxadiazole phenyl ring with an aminothiazole structure, whereas the head group is composed of guanidine moiety. These compounds include an electron-deficient phenyl ring, and this substitution may cause subsequent interactions with amino acids Cys533, His556, and Tyr566 at the end of the binding pocket. This indicates that guanidine-based compounds have cell permeability and potent inhibition of SphK1/2 activity. Also, it attenuates cellular S1P levels of U937 cells with no change in the level of sphingosine [127].

### SLP7111228

SLP7111228 is a guanidine-based inhibitor for SphK1 with Ki 48 nM [125]. Modification of SphK2 inhibitor SLP120701 through homologation with a single methylene moiety between the oxadiazole and heterocyclic ring provided a significant SphK1 selectivity in SLP7111228 [19]. Its chemical name is (S)-2-(3-(4- octylphenyl)-1, 2, 4-oxadiazol-5-yl) methyl) pyrrolidine-1 carboximidamide hydrochloride [117]. It induces the reduction of S1P by increasing phosphorylation level of Akt/ERK in U937 cells in addition to mice and rats models [117]. Administration of SLP7111228 in vivo causes depression of blood S1P levels [19].

### SLM6031434

SLM6031434 (2S)-2-[3-[4-(Octyloxy)-3-(trifluoromethyl)phenyl]-1,2,4-oxadiazol-5-yl]-1-pyrrolidine carboximidamide hydrochloride arises from alteration on SLR080811, incorporation of a meta-trifluoromethyl group on the internal phenyl ring, and the phenyl ring is attached to the lipophilic alkyl chain via an ether linkage [128]. SLM6031434 is an SphK2 inhibitor with K_i_ 370 nM. It causes a decline in cellular S1P concentration. SLM6031434 has an anti-fibrotic potential against the progressive renal fibrosis model in mice [129].

The hallmark features of renal fibrosis include inflammation and excessive extracellular matrix formation, which can ultimately result in functional insufficiency or kidney failure. Treatment with SLM6031434 in vivo increases the expression level of Smad7, a negative regulator of the pro-fibrotic TGFβ/Smad signaling cascade [130].

## Amidine-based SphK inhibitors

Amidine-based SphK inhibitors are other structural sphingosine analogs that inhibit the process of substrate binding to the SphKs domain [123]. This category includes VPC96077, and VPC96091, Table 1. If the hydroxyl group was considered responsible for phosphorylation, the more difficult to phosphorylate, would be more effective in suppressing the SphKs activity [123]. Furthermore, it was stated that amidine-based SphK inhibitors showed high selective activity in vitro towards SphK1 in the nanomolar scale.

### VPC96091

VPC96091, (2S)-1-(4-dodecylbenzoyl)-N'-hydroxypyrrolidine-2-carboximidamide, is characterized by a terminal α-substituted amino group linked with 4-alkyl phenyl via an amide bond [131]. It is produced after modification of L-alaninamide hydrochloride by dehydrating amide to nitrile and coupling of amidoxime to p-octylbenzoic acid along with reduction using dimethylformamide (DMF) and heating. It is an efficient and selective inhibitor with K_i_ values of 0.1 μM for SphK1 and 1.5 μM for SphK2 [132]. Selective inhibition of SphK1 by VPC96091, induces the reduction of epidermal growth factor (EGF), which drives S1P levels and then increases Akt/ERK phosphorylation in human leukemia U937 cells and mice model.

### VPC94075

VPC94075, [(S)-*N*-(1-amino-1-iminopropan-2-yl)-4-octylbenzamide hydrochloride is a weak inhibitor for the two SphK isoforms with IC_50_ of 55 μM for SphK1 and 20 μM for SphK2 through competition with sphingosine. It could reduce S1P and exert anti-proliferative activities [133]. VPC94075 is generated after hydrogenolysis of VPC96091 and further reduction of the N–O bond and tautomerization and rearrangement to give a more stable compound.

### Compound 28

Compound 28 is considered the most effective amido-derivatives inhibitors that are produced after modification of VPC45129 by adding amido group. It has favorable specificity towards SphK1, with Ki values of 0.3 μM and 6 μM for SphK1 and SphK2, respectively [134]. It causes a decrease in cellular S1P level, disrupting the sphingosine cycle and initiating cell cycle arrest.

### Compound 11

Compound 11 is considered the most compound that has high selectivity towards SphK1 with K_i_ 0.32 µM SphK1 and 8 µM SphK2 [135]. It is generated after the modification of 1-dodecene and the incorporation of an oxadiazole into the molecular scaffold. A unique cyclopropane ring torsional angle in compound 11 provides enhanced amidine presentation in the active site [136]. Compound 11 is the most representative one that has a 705-fold selectivity for SphK1 [19].

### Compound 1a

Compound 1a is considered an amidine-containing SphK1 inhibitor with K_i_ 0.1 µM for SphK1 and 1.5 µM for SphK2 [137]. Compound 1a is derived from l-proline. It can decrease cellular S1P in U937, Jurkat T lymphocytes, and SKOV3 cell cultures. It blocks S1P formation from Sphingosine [138]. Drug 1a competes with sphingosine in a concentration-dependent manner, however, it didn’t show an impact on cell viability when SphK1 is efficiently blocked [139]. Also, in vivo, it rapidly decreases the levels of circulating S1P upon blocking SphK1.

## Piperidine-based SphK inhibitors

Piperidine is a heterocyclic amine that consists of a six-membered ring containing five methylene bridges (–CH2–) and one amine bridge (–NH–) [140]. Piperidine analogs include compound 82, RB-005, SK1-5c (CAY10621), and Compound 1/2/3, Table 1.

### Compound 82

Compound 82 is a competitive inhibitor developed by modifying SKI-II through a structure-guided design approach [141]. It possesses inhibitory potency against SphK1, with IC50 values of 0.02 μM and 0.10 μM for SphK1 and SphK2, respectively [102]. Compound 82 decreases S1P production and sphingosine levels and increases ceramide concentrations in human breast and melanoma cell lines but has no impact on the growth of cancer cells [142]. The amino alcohol portions of compound 82, as well as two important aspartate residues in SphK1, establish hydrogen bonds that are crucial in the interaction between the compound and the target molecule [143]. Asp178 and Asp81 form hydrogen bonds with nitrogen on the piperidine ring and hydroxyl outside the piperidine ring of compound 82.

### RB-005

RB-005, 1-(4-octylphenethyl) piperidin-4-amine, is a specific SphK1 inhibitor with IC50 of 3.6 µM. RB-005 is a derivative obtained from the pathway of synthesizing FTY-720 from 4-octylphenylethanol [143]. This little modification in the tertiary amine structure is responsible for RB-005's ability to preserve SphK1 selectivity. RB-005 has high selectivity for SphK1 15.0 fold over SphK2 after comparing with RB-001- RB-022 [143]. RB-005 is characterized by an *n*-octylphenyl group linked in a 2-carbon tether to the nitrogen of 4-hydroxypiperidine. The hydroxyl group in the heterocyclic ring is important for inhibition of SphK1 [144]. Additionally, it can suppress ceramide synthase and promote SphK1 proteasome degradation in mice with hypoxic pulmonary hypertension [145].

### Compound 1/2/3

Compound 1/2/3 is a series of selective inhibitors of SphK1 derived from the framework of 2-piperidine thiazole [146]. The 4-position of the thiazole ring is commonly filled by a 5, 5, 8, 8-tetramethyl tetralin (Compound 1). On this basis, the structure is modified to produce greater diversity by joining piperidine at the 1-position, substituting piperidine with piperazine and different alkyl groups (Compound 2), or replacing the pentaryl group in previous patents with 2, 6-disubstituted pyridine (Compound 3) [20]. These compounds have therapeutic potential for rheumatoid arthritis (RA) and cancer. Scientists have declared that the IC_50_ value of these compounds is in the range of 1–1000 nM [117]. However, their mechanism of inhibition is still a mystery, whether competitive or non-competitive.

### SK1-5c (CAY10621)

SK1-5c, (2, 2-dimethyl-4S-(1-oxo-2 hexadecyn-1-yl)-1, 1-dimethylethyl ester-3-oxazolidinecarboxylic acid acts as a SphK1 inhibitor with IC_50_ = 3.3 μM, Ki = 3 μM. It possesses an anticancer capacity due to the suppression of tumor growth through reducing Akt signaling [147]. Also, it has an anticancer effect against colon cancer. Similarly, SK1-5c has an inhibitory impact against MDA-MB-231, and MCF-7 breast cancer cell lines in a dose-dependent manner [148]. SK1-5c effects include growth arrest, elevated apoptosis, and suppressed cell proliferation. On the other hand, in vivo treatment with SK1-5c caused a decline in serum-secreted S1P and serum-induced phosphorylation of both ERK1/2 and AKT, along with attenuated tumor growth in MDA-MB-231 xenograft in mice [149].

## Pyrrolidine-based SphK inhibitors

Pyrrolidine is the parent component of the pyrrolidine family that is characterized with a five-membered ring comprised of four carbon atoms and one nitrogen atom. Pyrrolidine-moiety was considered an efficient moiety for potent SphK inhibitors [117]. Pyrrolidine analogues include CHJ01, Compound 51, and PF-543, as shown in Table 1.

### CHJ01

CHJ01 is a synthetic analog of jaspine B, a hydrophytosphingosine resultant found in the marine sponges *Pachastrissa sp* and *Jaspis sp* [150]. Structural modifications were added to jaspine B to generate 2-epi-jaspine B, synthesized 17 compounds (YHR1-17), and recently produced hydrochloride salt CHJ01 [117]. CHJ01 is synthesized based on YHR1 after adding methanol and HCL. CHJ01 triggers a reduction of intracellular S1P levels and increases ceramide levels by inhibiting SphK1. The hydrochloride of CHJ01 exhibits potent inhibition against SphK1 with IC_50_ 8.64 μM. It also shows a distinctive therapeutic impact on RA via attenuation of inflammatory cytokines [150].

#### Compound 51

**Fig. 6:**
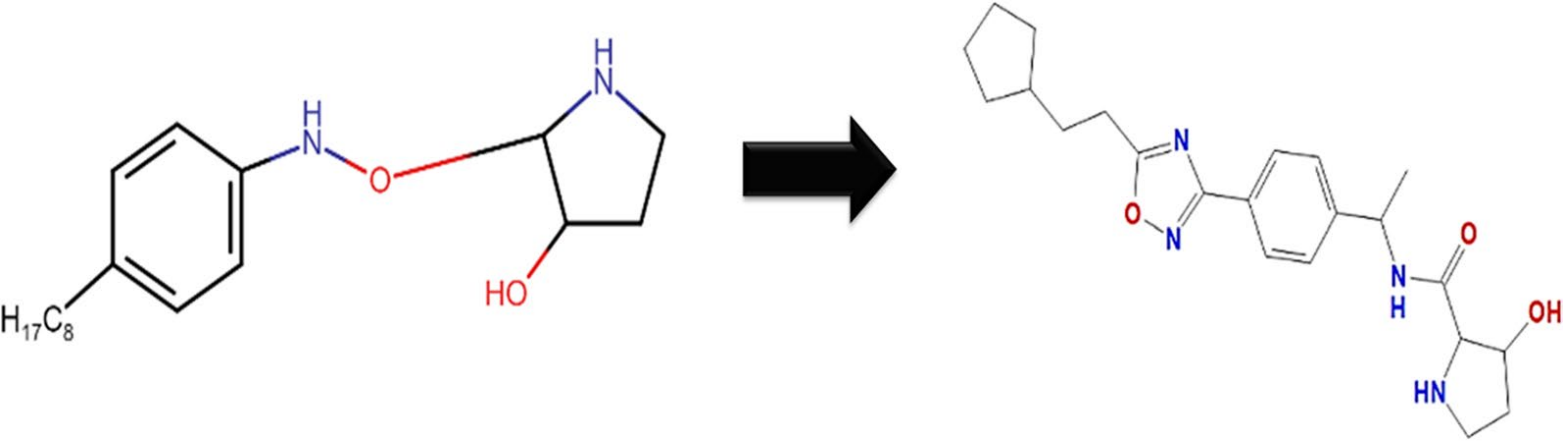
Generation of Compound 51 after modification of *N*-(5-alkyloxadiazol-3-yl) benzyl)-3-hydroxypyrrolidine-2-carboxamide scaffold (compound 4). The chemical structures used in the present illustration were drawn using ChemDraw Professional 21.0 software

Compound 51, (2S, 3S)-*N*-(S)-1-(4- (5-(2-cyclopentylethyl-1, 2, 4-oxadiazol-3-yl phenyl) ethyl hydroxypyrroli-dine-2-carboxamide, is considered a potent SphK1 inhibitor with the IC_50_ of 0.058 μM [151]. Compound 51 is generated after modification of *N*-(5-alkyloxadiazol-3-yl benzyl)-3-hydroxypyrrolidine-2-carboxamide scaffold (compound 4) (Fig. 6). The cyclopentenyl group linked to the oxadiazole ring enhances its activity and solubility. It displays moderate oral bioavailability, good internal clearance, and a favorable half-life in blood circulation [117].

### PF-543

**Fig. 7:**
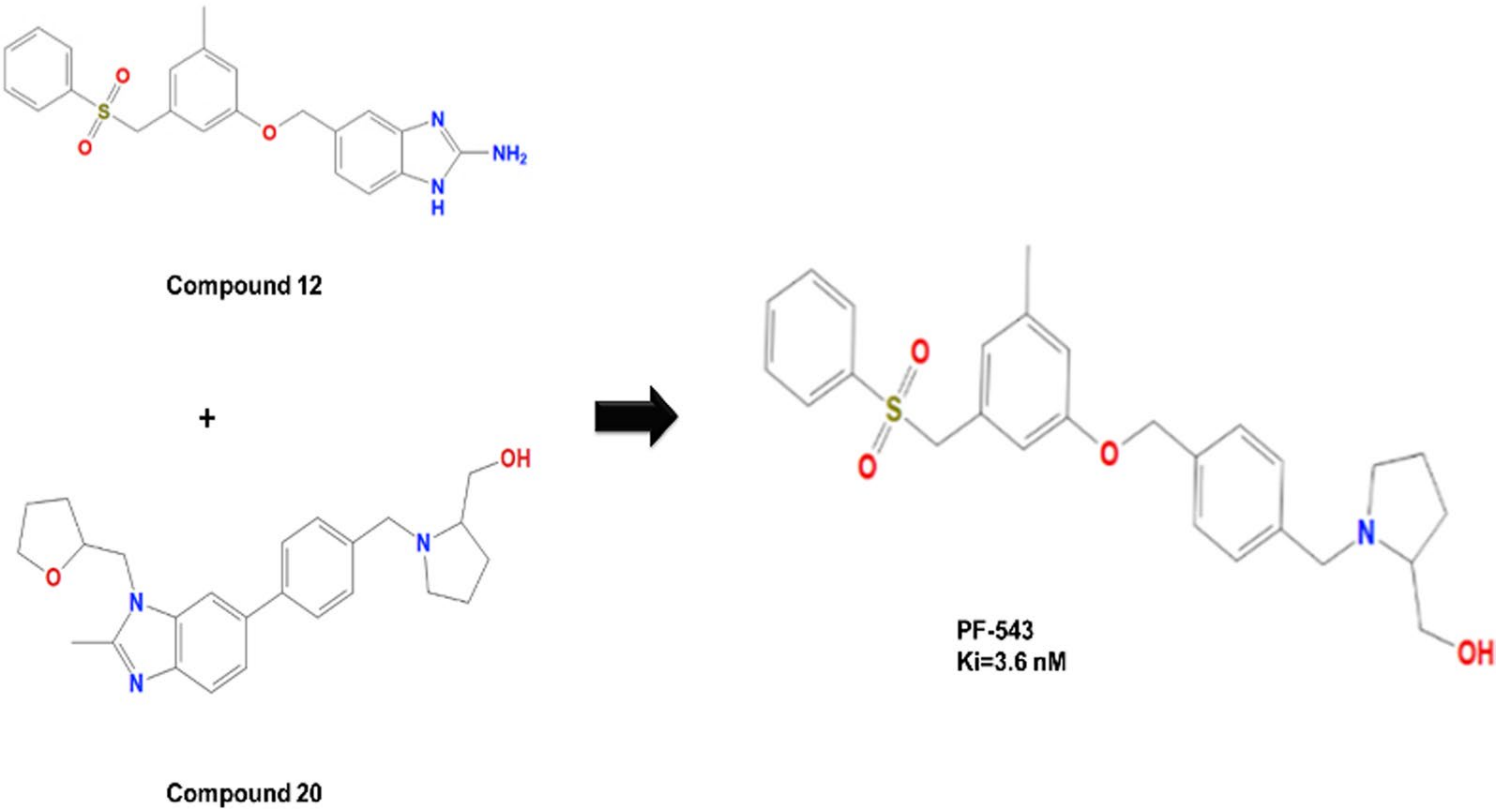
Combining fragments of two hits of molecules (12 and 20a) to generate PF543. The chemical structures used in the present illustration were drawn using ChemDraw Professional 21.0 software

PF-543, (R)-(1-(4–3-methyl-5 phenylsulfonylmethyl phenoxy) methyl benzyl pyrrolidin-2-yl methanol, is the most advanced SphK1 inhibitor with Ki of 3.6 nM. Once bound to SphK1, it causes conformational change and then proteasomal degradation of SphK1. Scientists discovered PF-543 by combining fragments of two hits (12 and 20a) or (5-3-(benzenesulfonyl) methyl]-5-methylphenoxy}. methyl)-1H-1,3-benzodiazol-2-amine, and 1-4-2-methyl-1-[(oxolan-2-yl)methyl-1H-1,3-benzodiazol-6yl-vphenyl methyl]pyrrolidin-2-yl methanol, respectively, (Fig. 7), [117]**.** The tail groups, such as 3-methyl group substitution, didn’t point out the importance of SphK1 inhibition, but the sulfonyl group was essential for selectivity. PF-543 was found to be an effective SphK1 inhibitor with a K_i_ of 3.6 nM. PF-543, in contrast, seems to have minimal impact on cellular ceramide levels while significantly reducing S1P and increasing sphingosine, which could explain its ineffectiveness in causing apoptosis [152].

Despite a significant reduction in the S1P/sphingosine ratio, PF543 did not influence the proliferation or survival of head and neck squamous cell carcinoma (HNSCC) cells. PF-543 acts as an anticancer agent and inhibits tumor growth of colon cancer through a cyclophilin D-mediated programmed-necrosis pathway, not an apoptosis pathway [153]. Conversely, PF543 pointed out substantial antiproliferative and cytotoxic effects in human colorectal tumor cells at concentrations of 2.5 µM or above, resulting in necroptosis. In animal trials, intravenous injection of PF543 decreased HCT-166 xenograft growth while significantly enhancing mice survival. Oral squamous cell carcinoma (SCC) cells were treated with PF543 at a dosage of 25 µM that decreased cell viability and triggered apoptosis, necrosis, and autophagy; however, cell survival is promoted. The inhibitory impact of PF543 on cell survival and proliferation at relatively high concentrations is thought to be due to its unintended effects on cellular enzymes, including SphK2. PF543 sensitizes breast cancer cells against 5-FU and doxorubicin during combination therapy [154].

### Naphthalene-based SphK inhibitors

Bicyclic aryl-based SphK inhibitors are fused bicyclic, including isoquinoline, naphthalene, quinazoline, quinoline, and indole, acting as sphingosine analogs. Among them, naphthalene-based compounds are considered one of the most studied compounds [154]. They exhibited a modification in the tail region, including SLC4011540, SLC5081308, SLC5091592, and SLC5111312 compounds, Table 1. The lipophilic-tail removal completely omitting the naphthalene-based SphK inhibitor's inhibitory activity, indicating the importance of the tail region to carry internal phenyl rings [155]. It was found that these analogs have a binding mode similar to sphingosine, allowing a significant competition effect.

### SLC5091592

SLC5091592 is considered one of the most potent naphthalene-based SphK inhibitors with Ki = 1.02 μM. SLC5091592 is a second-generation derivative of the SLR080811 scaffold [156]. Elevated SphK2 specificity is attained through the naphthyl moiety that enhances π-stacking interactions with Phe548 and van der Waals interactions with Cys533, Tyr566, and His556 in the binding pocket's tail region of SphK2. Screening of compounds including SLC5081308, SLC5091592, SLC5101463, SLC5121467, SLC5101465, SLC5101464 at 1 μM inhibitor concentrations with SphK1 and 0.3 μM with SphK2 results in Ki > 20 µM for SphK1, 1.02 µM ± 0.2 for SphK2, and selectivity fold > 20 for SphK2 [157]. It is composed of a 4-trifluomethylbenzyl ‘tail’, which is considered the reason for SLC5091592's substantial selectivity for SphK2 [127].

The molecule's length, particularly the activity, and selectivity of SphK2, appears to be linked to the length and optimal head-to-tail length (positive charge to terminal methyl group) of about ~ 18–21 atoms of the alkyl chain of naphthalene-based inhibitors, indicating a larger lipid binding pocket in SphK2 compared to SphK1 [158].

## Amino alcohol-based SphK inhibitors

Another group of sphingosine analog inhibitors is amino alcohol-based SphK inhibitors, which bear an amino alcohol head group and includes (S)-FTY720-vinylphosphonate, FTY720, FTY720-OCh_3_, and Sg-12, Table 1. The amino alcohol group targets SphK2 along with competing with sphingosine [130].

### SK1-I

SK1-I, (2R, 3S, 4E)-*N*-methyl-5-(4- pentylphenyl)-2-aminopent-4-ene-1, 3-diol), is widely used as a selective SphK1 inhibitor [159]. Its inhibitory mechanism involves competing with the substrate, as evidenced by its Ki value of 10 μM. SK1-I is generated from the replacement of the alkyl chain with a phenyl ring or substituting fluorine for the 3-hydroxyl group, yielding potent SphK inhibitors [160]. SK1-I reduces cellular S1P levels without changing levels of sphingosine or dihydrosphingosine, with an increase in total cellular ceramide and a decrease in sphingomyelin [161].

Therefore, it is used as an anticancer by reducing cancer cell S1P levels, inducing cancer cell apoptosis by inducing activation of caspases-3, and caspases-9, and reducing both hemangiogenesis and lymphangiogenesis. Recently, SK1-I promoted TP53 and expression of pro-apoptotic factors of downstream BCL_2_ by provoking autophagy and cancer cell death along with suppressing the proliferation of cancer cells within colon and breast carcinoma [162]. SK1-I acts as a chemosensitizing agent via activating apoptosis and decreasing ERK1/2 and Akt pro-survival signaling. Notably, owing to its high solubility and potent cytotoxic effect, SK1-I has been applied in vivo in animal disease models with cytotoxic impact in acute myeloid leukemia [163].

### DHS (Safingol)

The closest sphingosine analog is Safingol, the synthetic L-threo-stereoisomer of endogenous (d-erythro-) sphinganine. Its inhibitory mechanism involves competing with SphK1, as evidenced by its Ki value of 3–6 μM along with high ceramide levels and promoted apoptosis in several cell types [102]. It also acts as a lysosphingolipid protein kinase C (PKC) inhibitor that competitively interacts at the regulatory phorbol binding domain of PKC. It also acts as an apoptogenic agent accompanied by autophagy induction in a cancer cell line, such as the HCT-116 colon carcinoma cell line. However, the anticancer activity of Safingol is not confined to its anti-PKC action [155]. After treating MDA-MB-231 breast cancer cells and HT-29 colon cancer cells with 5–10 µM Safingol, there was activation for autophagy through altering AMP-activated protein kinase (AMPK) [164].

In addition, there are cellular changes were observed, such as down-regulation of anti-apoptotic agents (Bcl-xL) and up-regulation of apoptotic agents (Bax) expression levels which mediated ROS species resulting in necrotic cell death. After treating human oral SCC cells with different doses (25–50 µM), there was an increase within ROS species and down-regulating anti-apoptotic species that released endonuclease G into cytoplasm, inducing DNA fragmentation mediating apoptosis [165]. It can trigger autophagy in human colon tumor cells with 12 µM and subsequent ER stress and increased concentrations of endogenous dihydroceramide and dihydrosphingosine, along with the production of ROS species and cell death. Recently, safingol has been the primary efficient repressor used as an anticancer agent against solid tumors and leukemia [166]. It is used as an adjuvant drug combined with cisplatin in a patient with solid tumors.

### DMS

DMS, N, *N*-Dimethyl-d-erythro-sphingosine possesses an inhibitory effect on SphK1, with Ki of 30 µM. d-erythro-sphingosine is synthesized in four steps with a 33% overall yield from L-serine [123, 167]. DMS could inhibit tumor cell growth and promote apoptosis in several cancer types, including AML, chronic myeloid leukemia (CML), melanoma, colon, lung, prostate, breast, hepatoma, gastric, melanoma, epidermoid carcinoma, and neuroblastoma [168]. Also, it acts as a potent anticancer against A549 cells and human lung cancer cells. It inhibits cancer cell growth through suppressing SphK1 and nuclear factor-κB (NF-κB) p65 [169].

Furthermore, it decreases S1P with modulation of cellular ceramide levels and is able to increase intracellular Ca^+2^, mediating apoptosis. In athymic mice, DMS attenuated the proliferation of lung and stomach carcinoma in a dose-dependent manner and significantly reduced melanoma cell lung metastasis in syngeneic mice. Recently, S1P was considered as a new biomarker in food allergy in a clinical study [170]. Also, DMS is an active anti-inflammatory agent that reduces ovalbumin-induced airway hyper-responsiveness (AHR) and inflammation of the airway in mice sensitized to ovalbumin [171]. It reduces the number of eosinophils as well as the percentage of TNF-α, eotaxin, and chemokine ligand 2 (CCL2) in the bronchoalveolar lavage fluid [172].

### K145

K145, 3-(2-amino-ethyl)-5-[3-(4-butoxyl-phenyl)-propylidene]-thiazolidine-2,4dione, acts as a selective SphK2 inhibitor with Ki of 6.4 μM with competitive behavior with sphingosine [123]. K145 is generated from 4- butoxy-benzaldehyde followed by combining with Meldrum’s acid in the existence of piperidine followed by reduction to produce 3-(4-butoxy-phenyl)-propionaldehyde, then other reactions to produce K145 in a good yield [121]. It could decrease cellular S1P without affecting ceramide levels and decrease ERK1/2 and Akt signaling. Subsequently, this induces apoptosis with IC_50_ 4.30 μM in U937 cells treated with K145 [173].

### SG12 and SG14

**Fig. 8:**
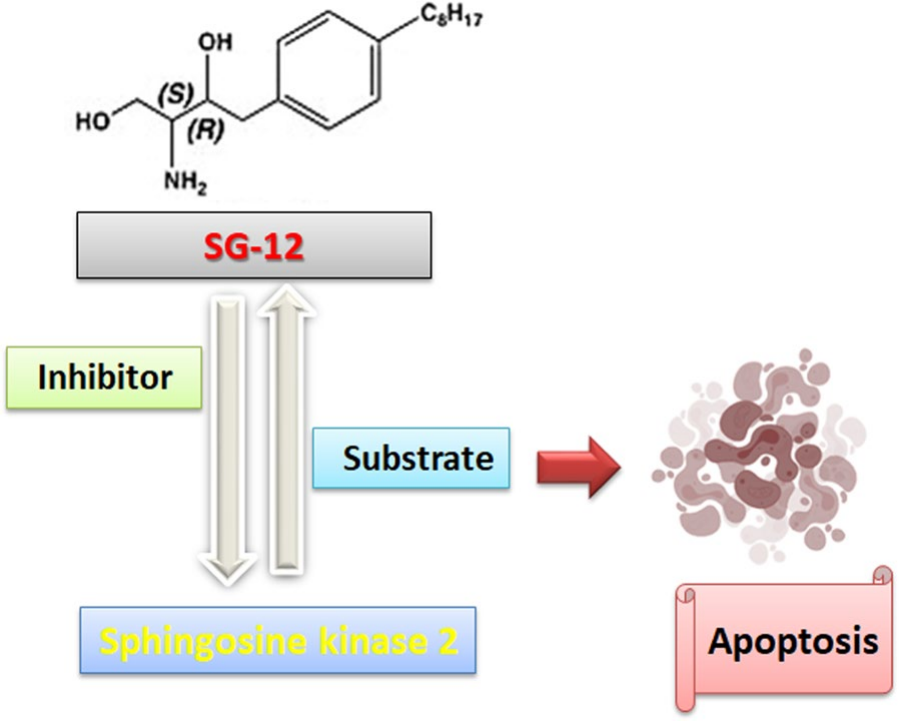
SphK2 phosphorylation promotes apoptosis via SG-12 and its inhibitory effect. The figure was drawn by using biorender https://www.biorender.com/

SG12 and SG14 are sphingosine analogs that act as selective inhibitors of SphK2 over SphK1. SG12 and SG14 have IC_50_ for SphK2 22 µM and 4 µM, respectivly. SG12 and SG14 were generated after modification of N, N-Dimethylsphingosine (DMS) [174]. R1 of DMS is substituted with the Octyl group; R2 and R4 are substituted with the O–H group to generate SG12 [175]. SG14 is produced after the substitution of R1 of DMS by H, the Pyrrolidine group substitutes R2, and R3 is replaced by the stearoyl group [176]. SG12 induces apoptosis in the murine B lymphoma-derived cell line A20/2J through the phosphorylation by SphK2 (Fig. 8).

After the screening of compounds including DMS, SG-1, SG-2, SG-3, SG-4, SG-5, SG-6, SG-7, SG-8, SG-9, SG-10, SG-11, SG-12, SG-13, SG-14, SG-15, and SG-16 towards SphK1/2 at concentration 50 µM [177]. It was found that SG12 and SG14 exhibited potent inhibitory effects against SphK2 with no effect on SphK1. SG-12 is considered an effective substrate for SphK2 with similar KM (5.5 μM) to sphingosine. SG12 induces phosphorylation of SphK2 resulting in inhibition and subsequent triggered apoptosis in the cancer cell line [175]. The level of SphK2 is up-regulated in the oncogenic environment, and SphK2 mRNA is up-regulated in colon and lung cancer cells [178]. Therefore, SG14 sphingosine analogs are considered promising therapeutic agents that inhibit the activity of SphK2 and induce apoptosis [33].

### Amgen 82

The 82nd compound (Amgen 82) exhibits inhibitory activity against both isoforms of SphK and has a remarkable pharmacokinetic profile. Amgen 2 possesses different selectivity of IC_50_ of 0.02 µM and 0.10 µM for SphK1 and SphK2, respectively [102]. Amgen 82 is generated by joining the structures of sphingosine and SKI-II to produce (2R,4S)-2-(hydroxymethyl) piperidin4-ol moiety and followed by a further modification to develop compounds such as Amgen 82 [141]. Amgen 82 induces cell death at higher concentrations. While therapeutic dosages could reduce S1P intracellular concentrations without affecting cell viability [116]. Amgen-82 possesses detergent-like physicochemical features that make the cell death effect need a greater amount to be administered [138]. Nevertheless, low S1P levels in the blood and treatment of Amgen-82 did not have any impact on the growth of the tumor in the xenograft mice model [179].

### 6ag/9ab/12aa

Through a sequence of sphingosine-1 modifications, powerful and new SphK1 inhibitors (6ag, 9ab, and 12aa) were obtained [151]. This modification includes replacing the amino diol headpiece of sphingosine with a serine amide. The carboxylic acid of serine increases affinity towards binding to lipophilic tail within SphK1, explaining the high inhibitory activity of these compounds towards SphK1 [117]. The compound 6ag showed nearly ten times greater activity upon incorporation of L-threonine as the polar headpiece. Furthermore, the S-enantiomer 9ab was nearly 40 times as compared to the R-enantiomer (50 nM vs. 2.2 μM). Thus, the stereochemistry of homoserine analogs was linked to a significant effect on activity [151]. Compound 12aa is a substantially more potent inhibitor with modification at the polar headpiece with 3-hydroxyproline.

Screening of compounds including 6aa, 6ab, 6ac, 6ad, 6ae, 6af, 6ag, 6ah, 6ai, 6aj, 6ak, and 6bl towards SphK1 showed that 6ag is the most potent inhibitor in the group with IC_50_ 0.65 μM. Also, Screening of compounds including 9aa, 9ab, 9ac, 9ad, 9ae, and 9bc towards SphK1 showed that 9ab has inhibition activity with IC_50_ 0.05 μM. Also, screening of compounds including 12aa, 12ab, 12ac, 12ba, and 12ca towards SphK1 showed that 12aa has inhibition activity with IC_50_ 0.062 μM [151]. The amide moiety was crucial for inhibitor strength, owning IC_50_ 0.65, 0.05, and 0.062 μM for 6ag/9ab/12aa, respectively, with more robustness as compared with DMS that has IC_50_ 24 μM [117]. Furthermore, none of these SphK1 active inhibitors demonstrated any activity towards SphK2 when screened at a concentration of 10 µM.

### FTY720 (Fingolimod) S1P receptor-independent

FTY720, (2-amino-2-[2-(4-octylphenyl) ethyl] propane-1,3-diol), inhibits SphK1 and SpjK2 with Ki 2 µM and 18.2 μM, respectively [180]. It is a synthetic sphingosine analog that is generated from a chemical modification of a natural product, myriocin (ISP-I). ISP-I is isolated from the culture broth of the fungus *Isaria sinclairii*. Interestingly, it is administered as a chemotherapeutic agent due to SphK1’s proto-oncogenic role [181]. Also, it mediates reactive oxygen species production, inducing apoptosis in the liver, prostate, and breast cancer treatment with IC_50_ 5–10 µM [182, 183]. It undergoes phosphorylation through SphK2, and as a result (FTY720-P) was formed, acting as an antagonist of four of the five S1PRs (excluding S1PR2). Inhibition of S1PR by p-FTY720 made drug-resistant colorectal cancer cells and tumors more susceptible to cetuximab [184]. Additionally, P-FTY720 acts as an immunosuppressant for recurrent multiple sclerosis and blocks T lymphocyte leakage in lymphoid tissues and down-regulation of S1PRs [185].

### (S)-FTY720-vinyl-Pn

(S)-FTY720 vinyl phosphonate is a novel SphK1 inhibitor with Ki 14.5 µM. (S)-FTY720 vinyl phosphonate is generated from the modification of FTY720 [186]. It is an uncompetitive inhibition. Its binding to the presumed allosteric site in SphK1 is dependent on the generation of the enzyme-sphingosine complex [187]. It possesses anti-cancer potency by inhibiting S1P-stimulated rearrangement of actin in MCF-7 cells [188].

### ROMe (R)-FTY720-OMe)

ROMe (R)-FTY720-OMe) is generated by replacing the hydroxyl group of FTY720 with a methoxy group [29]. It is a selective competitive inhibitor for SphK2 with Ki 16.5 μM, while the inhibitor failed to inhibit SphK1 [189]. Also, it attenuated SphK2 expression along with increased cleavage of PARP. SphK2 inhibition triggers apoptosis in HEK293 cells [189].

## Non-lipid-like small molecule SphK inhibitors

### Benzene sulfonamide based SphKs inhibitors.

Benzene sulfonamide-based SphKs inhibitors were synthesized through a structure-based approach to target SphKs ATP-binding pocket [19]. Docking was applied to bind these compounds into SphKs ATP-binding pocket and is supposed to generate close linking with N22, T54, S79, G82, L83, R24, G80, D81, and S112 of ATP-binding pocket within SphKs. The confirmation of the orientation binding was achieved through the assessment of its inhibition ability of SphKs ATP-binding pocket mutants through alanine mutagenesis. The compounds inhibition was reduced by ~ twofold, and ~ threefold through the T54A, L83A, R185A, and S112A mutations and the S79A, R24A, and R191A mutations, respectively [19]. These findings impacted the ATP-binding pocket of the SphKs inhibitor target was confirmed.

### MP-A08

Through structural homology modeling and in silico docking with small-molecule libraries, MP-A08 named as 4-Methyl-*N*-[2-[2-[(4-methylphenyl) sulfonyl] amino] phenyl]imino]methyl]phenyl] benzenesulfonamide was identified. It contains two benzene-sulfonamide groups joined by a benzylidene-aniline group [19]. The in silico docking study addressed a high ATP-competitive selectivity for the two SphK isoforms. It owns a higher affinity to SphK2 than SphK1 with Ki 6.9 μM and 27 μM, respectively [190]. It showed a weak off-target effect only on testis-specific serine kinase (TSSK) in high concentrations. MP-A08 acts as an anticancer agent because it induces apoptosis in cancer cell lines [102].

Due to its binding within the SphK1-ATP pocket, elevation in sphingosine and ceramide was obtained along with a decline in S1P expression levels. The inhibitor induces mitochondrial apoptosis [191]. Interestingly, it reduces tumor growth and promotes apoptosis when applied to the lung cancer model [117].

### SKI-II

SphK inhibitors contain several non-lipid small molecules such as SKI-I, SKI-II, SKI-III, and SKI-IV were identified by a high throughput screening method [77]. SKI-II (2-(p-hydroxyanilino)-4-(p-chlorophenyl) thiazole) is the most studied compound in its category, with high oral bioavailability and limited toxicity. Screening of a library consisting of 16,000 chemical compounds for inhibitors of human SphK at a fixed concentration of 10–25 µM revealed that compound SKI-II at 5 µg/ml inhibits SphK activity by 85% and serves as a prototype for non-lipid SphK inhibitors [192].

The lipid-binding pocket of SphK is the target of SKI-II, which acts as a competitive inhibitor without interfering with ATP-binding. SKI-II is observed to act as a dual inhibitor for SphK1/2 with a twofold selective for SphK2 (Ki = 7.9 μM) over SphK1 (Ki = 16 μM). Also, SKI-II can cause an indirect inhibition of dihydroceramide desaturase (DES1) with Ki = 0.3 μM during de novo synthesis of ceramide, resulting in the decrease of the cellular S1P [193]. SKI-II proved effective when applied to different disease models, including solid tumor cell lines and in vivo attenuation of breast adenocarcinoma cells. Also, it pointed out the significance of chronic myeloid leukemia [194].

Additionally, SKI-II was effective for the treatment, reduction, or prevention of multiple kinds of tumors such as lung cancer, acute myeloid leukemia, glioblastoma (GBM), hepatocellular carcinoma, prostate cancer, colon carcinogenesis, Merkel cell carcinoma, and leukemia [195–197]. Also, SKI-II was potent for many other diseases, such as pulmonary fibrosis, hyperalgesia and inflammation, diabetic nephropathy, white matter lesions (WMLs), meal virus infection, and acute radiation syndrome [198, 199].

### 11b

Compound 11b selectively inhibits SphK1, as evidenced by its IC50 value of 3.1 µM. It is synthesized from SKI-II as the first structure and PF-543 as a standard model for design based on molecular modeling [141]. During the synthesis and design of new inhibitors and after the screening of compounds 10-19b and 11-19a for SphK activity at 59.1 µM. Compound 11b had the most functionality in the whole series [200]. The structure was characterized by quinoline pharmacophore and naphthyl residue found in the hydrophobic [117]. However, a new linker was discovered that is responsible for the enhancement of efficiency by extending the structure that binds to the Asp178 residue of SphK1's active site, thereby ensuring the inhibition mechanism [141].

### SKI-I

SKI-I, (*N*-[(2-hydroxy-1-naphthyl) methylene]-3-(2-naphthyl)-1Hpyrazole-5-carbohydrazide) was discovered during the screening of a library containing 16,000 chemical compounds as an inhibitor of human SphK at a fixed concentration of 10–25 µM [201]. Screening revealed that compounds I (SKI-I) at 5 µg/ml inhibit SphK activity by 99%. SKI-I was considered a prototype for non-lipid SK inhibitors. It inhibits SphK1 competitively with IC_50_ of 1.2 μM [132].

Additionally, it inhibits SphK2 with comparable affinity and cross-reacts with ERK2, PKC, and PI3K. SKI-I was synthesized from starting compounds called 2-acenaphthene and dimethyl oxalate in the presence of sodium hydride and followed by various chemical steps to generate SKI-I as the final compound. SKI-I can decrease the cellular S1P levels as well as promote long-chain and very-long-chain ceramide. It also reduces the expression of cellular sphingosine levels in melanoma cells, which helps in the attenuation of cancer cell growth and induction of apoptosis.

It possesses potency as an anticancer agent against breast cancer cells, brain, cervical, lung, pancreatic, and melanoma cancer cell lines [202]. SKI-I had antitumor activities without toxicity in mice, however, it pointed out poor bioavailability. Therefore, it might not be suitable as a targeted therapy for breast cancer. Studies showed that SKI-I is involved in different diseases such as breast cancer, melanoma, human osteoblasts, type-2 diabetes, and human Embryonic Kidney Cell Survival [187].

### SKI-I-Asp

SKI-I-Asp is an aspirinyl derivative of SKI-I. It was proven that SKI-I-Asp has a better half-life than the original SKI-I and was suggested as a promising prodrug. SKI-I-Asp is generated by treating SKI-I with 2-acetoxybenzoyl chloride in the presence of a base followed by condensation hydrazide [203]. SKI-I-Asp has equal effectiveness at inhibiting SphK1 as the parent SKI compounds at a concentration < 1.25 µM but is less effective than the parent SKI compounds at higher doses of 5 µM. SKI-I-Asp has a cytotoxic effect on cancer cell lines, including U87MG, HeLa, H460, H226, A549, MDA-MB-231, and MCF-10A.

### SKI-178

SKI-178, (N′-[1-(3, 4 dimethoxyphenyl) ethylidene]-3-(4 methoxyphenyl)-1H-pyrazole-5 carbohydrazide), is considered an SK1-selective inhibitor that was designed through modifying the original SK inhibitor, SKI-I. SKI-178 was synthesized by replacing benzyl rings with phenyl rings; It pointed out an inhibitory impact on SphK1 in vivo and in vitro [102]. Additionally, SKI-178 was recognized as a dual inhibitor of both SphK1 and SphK2 [204]. SKI-178 is a SphK1 selective inhibitor with K_i_ = 1.33 μM along with an impact on SphK2 to induce apoptosis of AML in different mouse models [187]. SKI-178 induces induced mitosis and apoptosis via attenuation of pro-survival Bcl-2 family and cyclin-dependent kinase 1 (CDK1) activation. Therefore, it can act as an anticancer agent against breast cancer cell lines.

### CB5468139

CB5468139 is an ATP-competitive inhibitor for SphK1 with K_i_ 10–15 μM. It inhibits the proliferation of A498 adenocarcinoma cells by decreasing cellular S1P levels and increasing ceramides along with the induction of autophagy within the cancer cell line [29].

## SphK inhibitors from natural sources

### Peretinoin

Peretinoin is one of the retinoid acids (retinoids), a byproduct, and an analog of vitamin A. It is a synthetic polyprenoic acid with retinoid-like characteristics that binds to the cellular retinoic acid-binding protein [102]. Researchers discovered that it is a potent chemotherapeutic agent in cancer treatment. After treatment of Huh-7 cells with peretinoin, inhibition of SphK1 promoter activity by blocking overexpression of SP1R was obtained. Interestingly, peretinoin inhibits hepatocarcinogenesis by lowering SphK1 mRNA levels [205].

### Hispidulin

Hispidulin is considered a phenolic flavonoid compound and potent SphK1. It was isolated mainly from *S. Involucrata* with the capacity to inhibit the activity of SphK1 by Ki 2.71 μM [102]. Also, it induces ceramide accumulation, thus leading to apoptosis of the cancer cells. Its chemical name is (4′, 5, 7-trihydroxy-6-methoxyflavone). It exerts a highly cytotoxic effect on cancer cells such as renal cell carcinoma, acute myeloid leukemia, and gallbladder cancer, HCC cell lines SMMC7721 and Bel7402. Also, it mediates apoptosis through activating caspase 3 [206].

Furthermore, it inhibits cell migration and invasion by suppressing the expression of matrix metalloproteinases (MMP-2, MMP-9) and enhancing the expression level of tissue inhibitor of metalloproteinase-3 (TIMP-3). Furthermore, it activates peroxisome proliferator-activated receptor γ (PPARγ) signaling pathway, which predominantly promotes the cytotoxicity of cancer cells [206].

### Icaritin

Icaritin (IC-162) is extracted from the hydrolysis of traditional Chinese herbal medicine *Epimedium sp*. Also, it is considered a SphK1 inhibitor with Ki 8.13- 18 μM [207]. It inhibits the proliferation of breast cancer cells and PC-3 prostate cancer cells by stimulating the ERK signaling pathway [208]. It exerts anti-HCC activity by a natural prenyl flavonoid Icaritin. It can also limit the activity of SphK1 in HCC cells, resulting in the production of pro-apoptotic ceramide and triggering of JNK1 [102].

### Balanocarpol

Balanocarpol is extracted from dried leaves of H. dryobalanoides and is considered an SphK1 inhibitor. As a dimer of resveratrol, it can down-regulate SphK1 expression and activity [155]. It is a sphingosine competitive inhibitor of SphK1 with inhibition concentration (Ki) 160 ± 40 μM [117]. Also, it triggers apoptosis in the prostate cancer cell [209].

### Pristimerin

Pristimerin is a triterpenoid that occurs in nature acting as a potent inhibition of SphK1 with Ki 0.2–4 μM. Its chemical name is 20α-3-hydroxy-2-oxo-24-nor-friedela-1-10, 3, 5, 7-tetraen-carboxylic acid-29-methylester [102]. It possesses anticancer properties hindering the growth of cancer cell lines such as glioma, leukemia, breast, lung, and prostate cancer cell lines through inhibiting NF-kB activity [210]. In hypoxic PC-3 cells, pristimerin can reduce HIF-1α, SphK-1 expression, and phospho-AKT/GSK3, along with lowering VEGF synthesis in hypoxic PC-3 cells [211]. HIF-1α accumulation is suppressed by blocking the activity of SphK1 promoting antioxidant capacity in PC-3 cells under hypoxia [212].

### Suramin

Suramin is a synthetic medicine antagonist of S1P receptors and is considered an SphK1 inhibitor with Ki 130 to 3715 μM [213]. It binds to the S1PR3 receptor, which is useful in the treatment of renal fibrosis disease [214]. It has an effective cytotoxic effect against human lung cancer cell lines [215]. Also, it decreases the expression of α-SMA and collagen deposition, along with a decreased level of hydroxyproline, thus ameliorating hepatic fibrosis induced by BDL [102].

### Ellagic acid

Ellagic acid (EA) is a natural polyphenol compound isolated mainly from a variety of fruits and vegetables and can act as an SphK1 inhibitor with Ki 0.74 ± 0.06 μM [216]. Its chemical name is 6,7,13,14-tetrahydroxy-2,9 dioxatetracyclo [6.6.2.04,16.011,15] hexadeca-1(15),4,6,8(16),11,13-hexaene-3,10 dione. It possesses an inhibitory impact against SphK1 due to its interaction with the nucleotide-binding site, lowering ATP accessibility and SphK1 catalytic activity [102]. EA pointed out induction for apoptosis along with AMP-activated protein kinase (AMPK) and reduction of HIF-1α in lung cancer cells. EA is used to inhibit tumor growth by reducing cell growth and damaging mitochondria [217, 218]. As a result, targeting SphK1 with EA to tip the sphingolipid rheostat towards pro-apoptotic ceramide.

### Epigallocatechin-3-gallate (EGCG)

EGCG is a natural product extracted from dried fresh leaves of *Camellia sinensis* with an inhibitory potency against SphK1 with Ki 75 µM. Its chemical name is (2R,3R)-5,7-Dihydroxy-2-(3,4,5-trihydroxyphenyl) -3,4-dihydro-2H-1-benzopyran-3-yl 3,4,5-trihydroxybenzoate. It triggers acid sphingomyelinase (ASM) activation, leading to ceramide accumulation and apoptotic cell death in cancer cells [102]. In multiple myeloma cells, activation of ASM by targeting 67-kDa laminin receptors (67LR) disrupts lipid rafts and suppresses receptor tyrosine kinase (RTK) activation. Multiple myeloma cells have much higher levels of SphK1, a negative regulator of ceramide accumulation with anti-apoptotic effects [219].

The apoptotic impact of EGCG, was enhanced when SphK1 was silenced [220]. Furthermore, through suppression of RTK phosphorylation and activation of death-associated protein kinase 1, the SphK1 inhibitor safingol synergistically sensitized EGCG-induced proapoptotic cell death and tumor suppression in multiple myeloma cells (DAPK1) [220]. The combination of EGCG/safingol suppresses viable cell numbers in chronic lymphocytic leukemia cell lines.

### B-5354C/F-12509a

F-12509a and B-5354C are produced by extraction from *Trichopezizella barbata* and a new marine bacterium, respectively. F-12509a suppresses SphK1 with an inhibition concentration (Ki) of 18 μM [102]. It contains a sesquiterpene moiety that reduces sphingosine levels once bound to the active site of SphK1 along with increased level of ceramide and attenuated S1P. The B-5354C is an ester of 4-amino-3-hydroxybenzoic acid with a long chain of unsaturated alcohol [117]. It inhibits SphK1 with a concentration of Ki of 12 μM. The type of inhibition varies among both inhibitors F-12509a is competitive inhibition, while B-5354C shows non-competitive inhibition.

### Pachastrissamine

Pachastrissamine (PA) is an anhydrophytosphingosine natural product isolated mainly from a sponge, *Pachastrissa sp*. It is one of the most naturally potent inhibitors of SphK2 with low Ki 4.6 μM [102]. It has an effective anticancer effect and induces apoptosis against cancer cells such as A549 and melanoma cells. It leads to the blocking of ERK and FOXO3 phosphorylation in melanoma cells, but its selectivity, bioavailability, and feasibility for large-scale production remain unclear.

## Computational and preclinical studies of SPHK1 inhibitors

In this review, we comprehensively performed a molecular docking analysis for all publicly available SphK1 inhibitors to gain further understanding of their potential effect against SphK1 and their relation to the responsiveness and sensitivity of the cancer cells towards drugs. Docking was performed using the Molecular Operating Environment software (MOE, 2015.10) and BIOVIA Discovery Studio Visualizer [221].

**Fig. 9:**
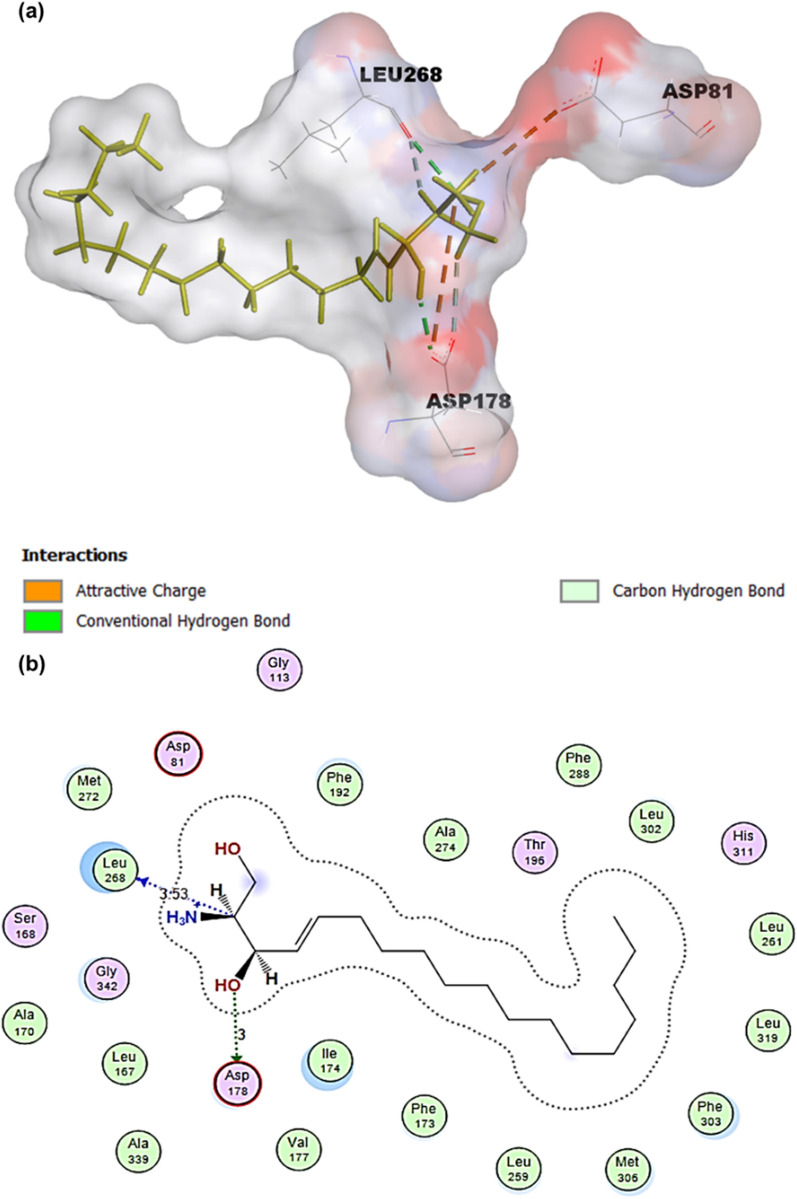
**a**, **b** 3D and 2D molecular docking results, respectively, for the co-crystallized of (2S,3R,4E)-2-aminooctadec-4-ene-1,3-diol** (**SQS) in SphK1. **a** Alkyl and pi-alkyl were removed, and the surface was optimized to atom charge. **b** 2D interaction diagram showing SQS docking pose interactions with the enzyme, including two amino acids ASP 178 and LEU 268 through attractive charge and conventional hydrogen bond, respectively. The figure was drawn by using MOE.2015 and Discovery Studio Visualizer

The reactions of the substances with critical amino acids or protein hot spots were also documented following a previously reported procedure [221–223]. The Protein Data Bank (PDB) is the source to provide target proteins with their 3D structures. For SphK1 inhibitors, the terminal domain co-crystallized with (2S,3R,4E)-2-aminooctadec-4-ene-1,3-diol (SQS) in the active site as an inhibitor (PDB ID: 3VZB) used for molecular docking, (Fig. 9). All structure minimizations were conducted till RMSD gradient of 0.05 kcal∙mol^−1^ Å^−1^ with *MMFF94x* force field and partial charges were automatically calculated. Additionally, all water molecules were removed from compounds, and then SphK1 was prepared for docking using *Protonate 3D* protocol in MOE with default parameters. The co-crystalized ligand (SQS) was used to determine the binding site for docking simulation. *Triangle Matcher placement* method and *London dG* scoring function were implemented for both docking and scoring. The docking protocol was first validated by self-docking of the co-crystallized ligand in the vicinity of the binding site of the protein. Then, the validated docking protocol (RMSD < 2) was used to study the ligand-receptor interactions at the protein binding site for the reported inhibitors to predict their binding mode and binding affinity.

**Fig. 10:**
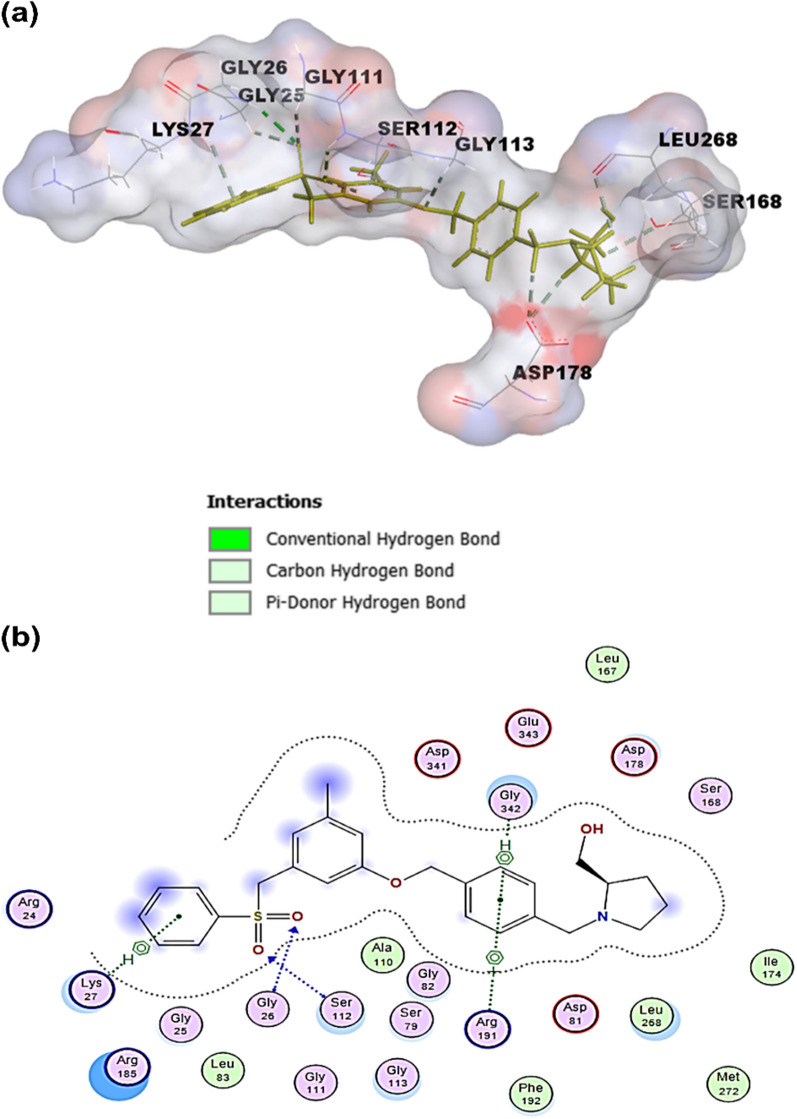
**a**, **b** 3D and 2D molecular docking results, respectively, for the reference molecule (PF-543) in SphK1. **a** Alkyl and pi-alkyl were removed, and the surface was optimized to atom charge. **b** 2D interaction diagram showing PF-543 docking pose interactions with the enzyme, including GLY26, and SER112 via conventional hydrogen bond. The figure was drawn by using MOE.2015 and Discovery Studio Visualizer

**Fig. 11:**
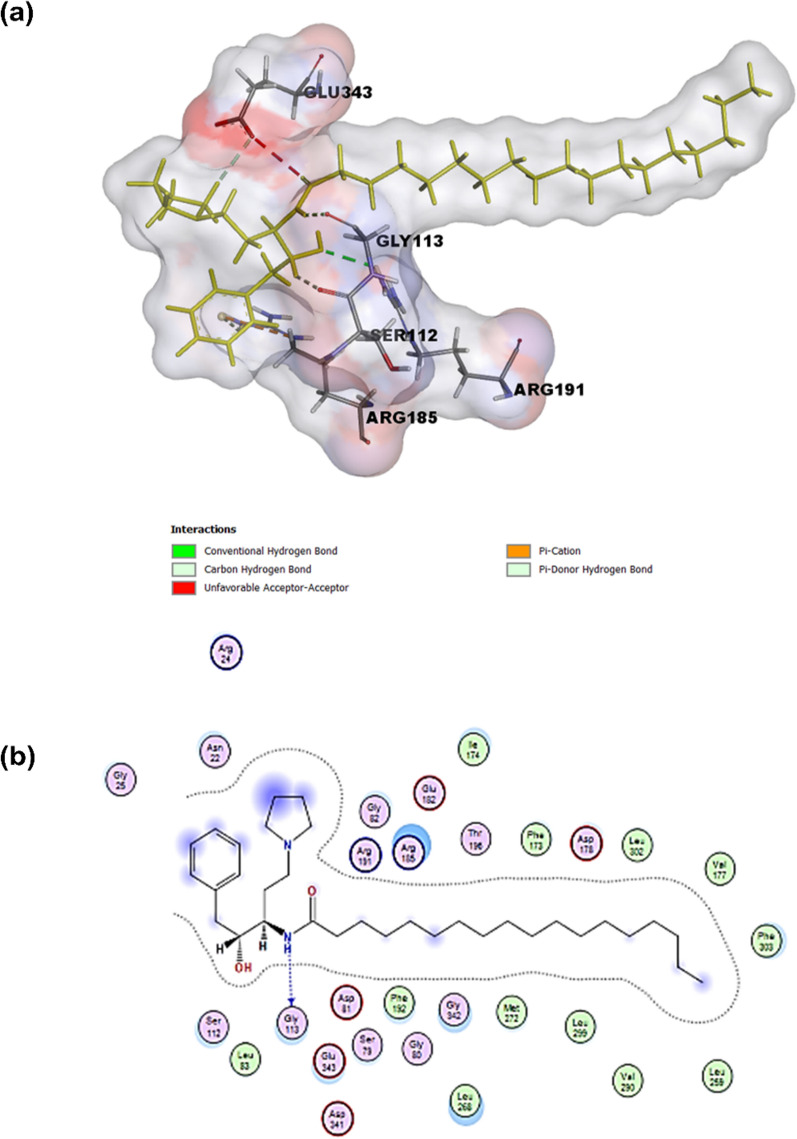
**a**, **b** 3D and 2D molecular docking results, respectively, for the inhibitor SG14 in SphK1. **a** Alkyl and pi-alkyl were removed, and the surface was optimized to atom charge. **b** 2D interaction diagram showing SG14 docking pose interactions with the enzyme, including GLY113 and ARG191, via conventional hydrogen bond. The figure was drawn by using MOE.2015 and Discovery Studio Visualizer

The inhibitory activity of the tested substances was compared to the most potent SphK1 inhibitor (Additional file 1: Figs. S1, S2, S3, Table 1), PF-543, through computational analysis-based investigations (Fig. 10) [224]. The plausible modes of binding between these substances and their target binding sites were determined to achieve this. SG14 (docking score; *S* =  − 11.4135 kcal/mol) was found to exhibit the most significant inhibitory activity in the group (Fig. 11, Table 1), with higher potency compared to the template co-crystalized ligand SQS (*S* =  − 8.0423 kcal/mol) and the reference molecule PF-543 (*S* =  − 8.9563 kcal/mol).

SG14 interacted with the SphK1 active site via hydrogen bonds by G113 and R191. Also, SLC4011540 (docking score; *S* =  − 9.3994 kcal/mol) was found to have the highest inhibitory activity within the group (Additional file 1: Fig. S1, Table 1), which was also 2nd higher molecule than those of SQS. Furthermore, SK1-5c (docking score; S =  − 8.9416 kcal/mol) had the same potent inhibitory effect as PF-543. On the other hand, other molecules didn’t show any interactions with the active site of SphK1, including SLR080811, SLM6031434, SLC5111312, SLP120701, SLC5091592, VPC96091, and VPC94075. Based on the docking simulations, it can be concluded that SG14, SLC4011540, and SK1-5c can effectively inhibit SphK1 and are, therefore, considered potent drugs to improve the sensitivity of cancer cells towards chemotherapies.

## Conclusion

Cancer drug resistance is still a problematic conundrum in the context of chemo/radiotherapy. It hinders several drugs’ effects and allows for several contributing resistance machineries to omit treatment efficacy and orient cells to a worse profile. S1P, and SphK1 overexpression are considered hallmarks of several carcinomas with involvement in several chemoresistance machineries. Thus, suppression of SphK1 might enhance the sensitivity of several drugs against cancer. Our team overviewed SphK1 expression among different cancers, several resistance processes, S1P metabolism, S1P transport, S1P signaling, and SphKs inhibitors, with molecular docking for up-to-date all publicly available SphK1 inhibitors. We addressed substantial computational inhibitory robustness among SG14, SLC4011540, and SK1-5c on SphK1. We here provide a preliminary pipeline to fight against cancer drug resistance. Also, several studies are required to validate SphK1 inhibition in the course of elimination of cancer drug resistance protocols.

### Supplementary Information

**Additional file 1: Fig. S1**. 2D diagrams of the reported inhibitors **(A)**. LCL351, **(B).** SLC4011540, **(C).** Compound 28, **(D).** Compound 1a, **(E).** Compound 82, **(F).** RB-005, **(G).** SK1-5c, (**H).** Compound 51 (SK1-IN-1), **(I).** PF-543, **(J).**SK1-I, **(K).** DHS (Safingol), **(L)**DMS, **(M)** K145, **(N)** SG12, **(O)**SG14, **(P)**Amgen 82, **(Q)** FTY720, **(R)** (S)-FTY720 vinyl phosphonate, **(S)** ROMe (R)-FTY720-OMe) showing their interaction with the key amino acids in the binding site for SPK inhibitors with the active site of Sphingosine Kinase 1 (3VZB). The figure was drawn by using MOE.2015. **Fig. S2**. 2D diagrams of the reported inhibitors **(A)**. MP-A08, **(B).** SKI-II, **(C).** 11b, **(D).** SKI-I, **(E).** SKI-I-Asp, **(F).** SKI-178, **(G).** SK-F, (**H).** Opaganib ABC294640, **(I).** ABC294735, **(J).** CB5468139, **(K).** ST-1803 shows their interaction with the key amino acids in the binding site for SPK inhibitors with the active site of Sphingosine Kinase 1 (3VZB). The figure was drawn by using MOE.2015. **Fig. S3. 2D** diagrams of the reported inhibitors **(A)**. Pachastrissamine (jaspine B), **(B)**. F-12509a, **(C)**. B-5354C, **(D)**. Balanocarpol, **(E)**. Icaritin, **(F)**. Hispidulin, **(G)**. Peretinoin, **(H)**. Pristimerin, **(I).** Suramin, **(J)**. Ellagic acid (EA), **(K)**. Epigallocatechin-3-gallate (EGCG) shows their interaction with the key amino acids in the binding site for SPK inhibitors with the active site of Sphingosine Kinase 1 (3VZB). The figure was drawn by using MOE.2015.

## Data Availability

Not applicable.

## Abbreviations

ASM: Acid sphingomyelinase
ABC: ATP-binding cassette transporter
CFTR: Cystic fibrosis transmembrane regulator
CERT: Ceramide transfer protein
CFTR: Cystic fibrosis transmembrane regulator
Cer: Ceramide
gln493: Glutamine493
miRNAs: Micro RNAs
MDR: Multidrug resistance
NSCLC: Non-small-cell lung cancer
lncRNAs: Long noncoding RNAs
SM: Sphingomyelin
S1P: Sphingosine-1-phosphate
Sph: Sphingosine
SPNS2: Spinster 2 transporter
TNFa: Tumor necrosis factor-a
WMLs: White matter lesions
