# Supplementary material for: The emerging roles of sphingosine 1-phosphate and SphK1 in cancer resistance: a promising therapeutic target
Source: Cancer Cell Int. 2024 Feb 28;24:89. doi: 10.1186/s12935-024-03221-8 (PMC10903003; doi:10.1186/s12935-024-03221-8)
Supplement: Supplementary file 1 — Additional file 1: Fig. S1. 2D diagrams of the reported inhibitors (A). LCL351, (B). SLC4011540, (C). Compound 28, (D). Compound 1a, (E). Compound 82, (F). RB-005, (G). SK1-5c, (H). Compound 51 (SK1-IN-1), (I). PF-543, (J).SK1-I, (K). DHS (Safingol), (L)DMS, (M) K145, (N) SG12, (O)SG14, (P)Amgen 82, (Q) FTY720, (R) (S)-FTY720 vinyl phosphonate, (S) ROMe (R)-FTY720-OMe) showing their interaction with the key amino acids in the binding site for SPK inhibitors with the active site of Sphingosine Kinase 1 (3VZB). The figure was drawn by using MOE.2015. Fig. S2. 2D diagrams of the reported inhibitors (A). MP-A08, (B). SKI-II, (C). 11b, (D). SKI-I, (E). SKI-I-Asp, (F). SKI-178, (G). SK-F, (H). Opaganib ABC294640, (I). ABC294735, (J). CB5468139, (K). ST-1803 shows their interaction with the key amino acids in the binding site for SPK inhibitors with the active site of Sphingosine Kinase 1 (3VZB). The figure was drawn by using MOE.2015. Fig. S3. 2D diagrams of the reported inhibitors (A). Pachastrissamine (jaspine B), (B). F-12509a, (C). B-5354C, (D). Balanocarpol, (E). Icaritin, (F). Hispidulin, (G). Peretinoin, (H). Pristimerin, (I). Suramin, (J). Ellagic acid (EA), (K). Epigallocatechin-3-gallate (EGCG) shows their interaction with the key amino acids in the binding site for SPK inhibitors with the active site of Sphingosine Kinase 1 (3VZB). The figure was drawn by using MOE.2015. [file 12935_2024_3221_MOESM1_ESM.docx]

Additional file

**“The emerging roles of sphingosine 1-phosphate and SphK1 in cancer resistance: A promising therapeutic target"**

Samar Sami Alkafaas**^1*^**^†^, Mohamed I. Elsalahaty**^2*^**^†^, Doha Mohamed**^2^**, Mustafa Ali Radwan**^2^**, Sara Samy Elkafas**^3,4^**, Samah A Loutfy**^5,6^**, Rami M. Elshazli**^7^**, Narjes Baazaoui**^8^**, Ahmed Ezzat Ahmed**^9^**, Wael Hafez**^10^**, Mohanad Diab**^11^**, Mohamed I. Sakran**^2,12^**, Mohamed T. El-Saadony**^13^**, Khaled A. El-Tarabily**^14^**, Hani K. Kamal**^15^**, Mohamed Hussin**^1^**

**^1^**Molecular Cell Biology Unit, Division of Biochemistry, Department of Chemistry, Faculty of Science, Tanta University, 31527, Egypt.

**^2^**Biochemistry Division, Department of Chemistry, Faculty of Science, Tanta University, Tanta, 31527, Egypt

**^3^**Production Engineering and Mechanical Design Department, Faculty of Engineering, Menofia University, Menofia, Egypt.

^4^Faculty of Control System and Robotics ITMO University, Saint-Petersburg, Russia.

**^5^**Virology and Immunology Unit, Cancer Biology Department, National Cancer Institute, Cairo University, Cairo, Egypt.

**^6^**Nanotechnology Research Center, British University, Cairo, Egypt

**^7^**Biochemistry and Molecular Genetics Unit, Department of Basic Sciences, Faculty of Physical Therapy, Horus University—Egypt, New Damietta 34517, Egypt

^8^Biology Department, College of Sciences and Arts Muhayil Assir, King Khalid University, Abha 61421, Saudi Arabia

^9^Department of Biology, College of Science, King Khalid University, Abha 61421, Saudi Arabia

**^10^**NMC Royal Hospital, 16th Street, Khalifa, Abu Dhabi 35233, United Arab Emirates; Medical Research Division, Department of Internal Medicine, The National Research Centre, Cairo 11511, Egypt.

**^11^** Burjeel Hospital Abu Dhabi, United Arab Emirates

**^12^** Biochemistry Department, Faculty of Science, University of Tabuk, Tabuk 47512, Saudi Arabia.

**^13^**Department of Agricultural Microbiology, Faculty of Agriculture, Zagazig University, Zagazig, 44511, Egypt.

**^14^**Department of Biology, College of Science, United Arab Emirates University, Al-Ain, 15551, United Arab Emirates.

**^15^**Anatomy and histology, Faculty of Pharmacy, King Abdulaziz University, Jeddah 21589, Saudi Arabia.

**Correspondence: Samar Sami Alkafaas* [*samar.alkafas@science.tanta.edu.eg*](mailto:samar.alkafas@science.tanta.edu.eg)*;* [*samarsamy2017@yahoo.com*](mailto:samarsamy2017@yahoo.com) *;Mohamed I. Elsalahaty:* [*mohamed.elsalahaty@science.tanta.edu.eg*](mailto:mohamed.elsalahaty@science.tanta.edu.eg) *†The authors are equally contributed to the work*

| **(A)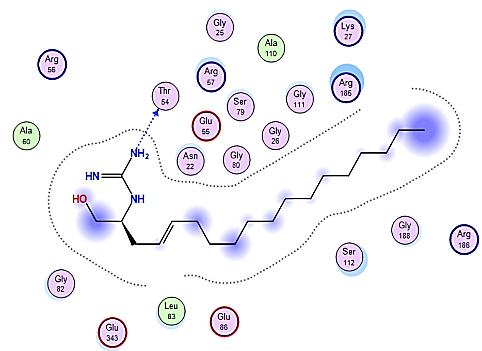** | **(B)**  **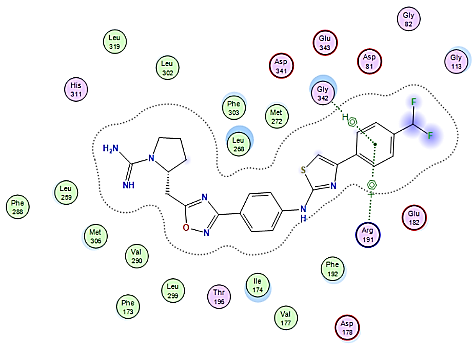** | **(C)**  **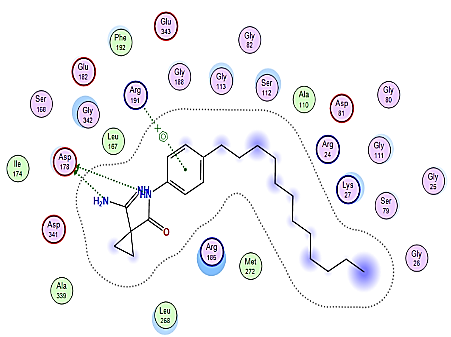** |
| --- | --- | --- |
| **(D)**  **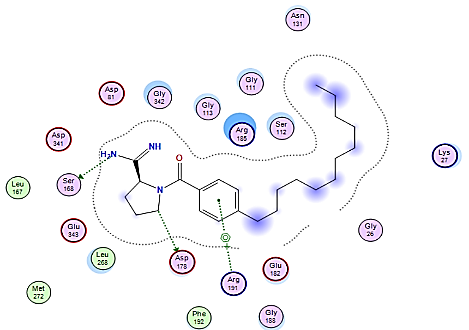** | **(E)**  **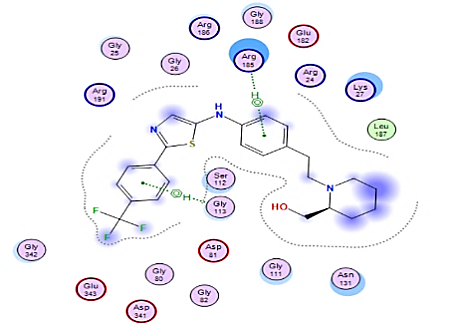** | **(F)**  **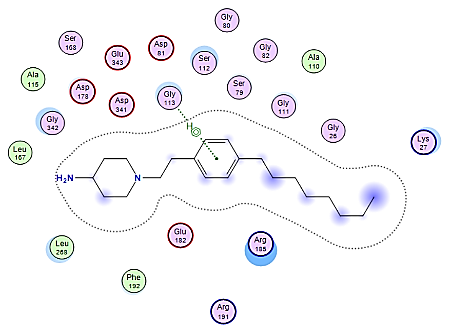** |
| **(G)**  **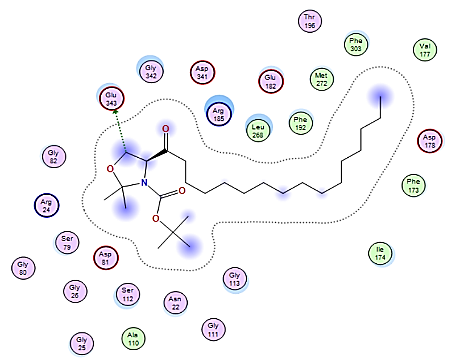** | **(H)**  **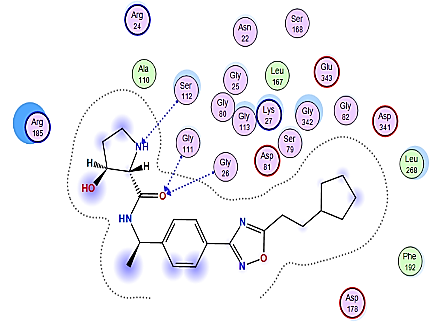** | **(I)**  **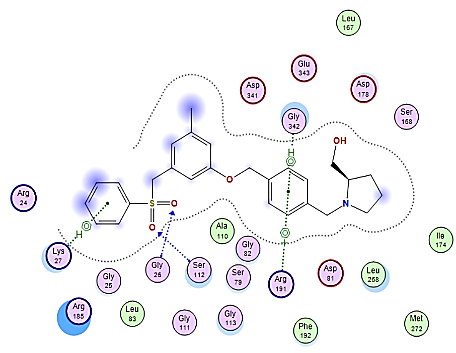** |
| **(J)**  **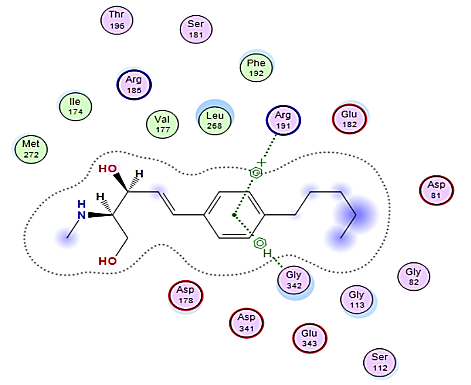** | **(K)**  **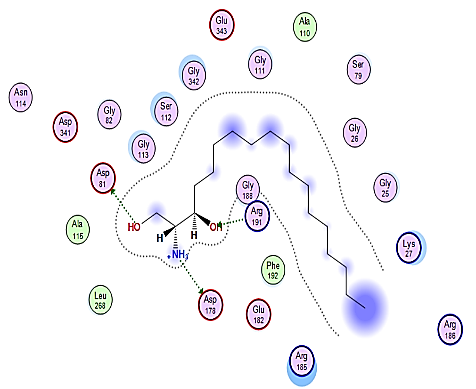** | **(L)**  **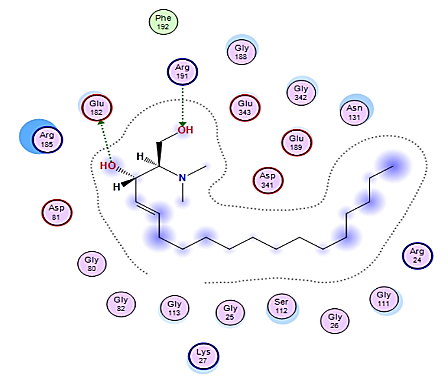** |
| **(M)**  **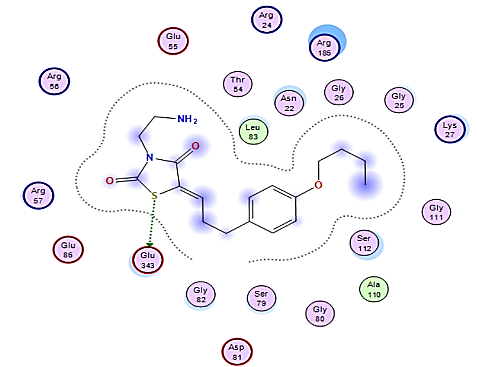** | **(N)**  **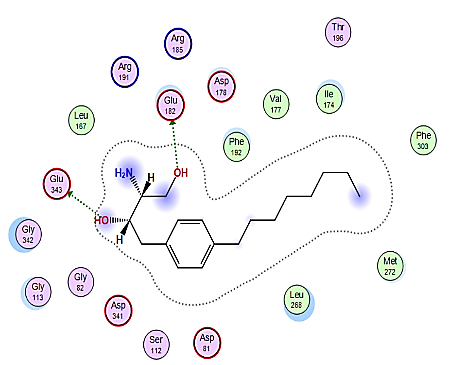** | **(O)**  **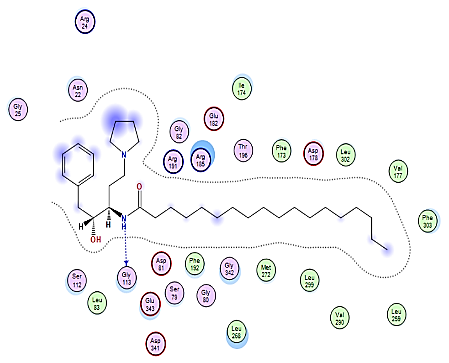** |
| **(P)**  **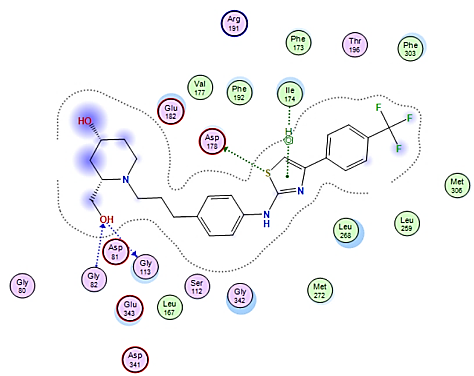** | **(Q)**  **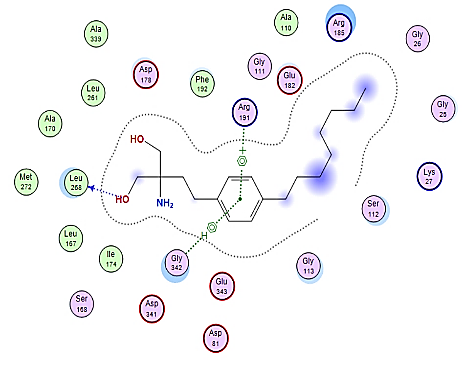** | **(R)**  **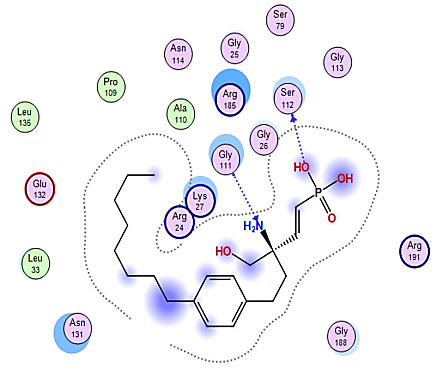** |
| **(S)**  **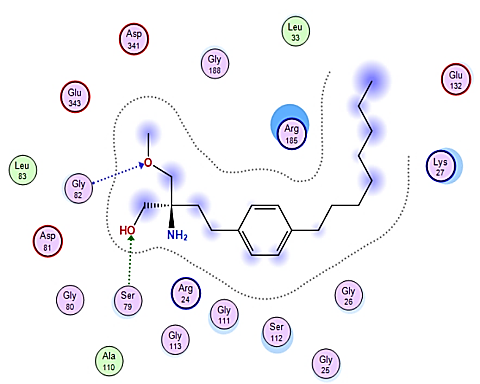** |  |  |

**Fig. S1**. 2D diagrams of the reported inhibitors **(A)**. LCL351, **(B).** SLC4011540, **(C).** Compound 28, **(D).** Compound 1a, **(E).** Compound 82, **(F).** RB-005, **(G).** SK1-5c, (**H).** Compound 51 (SK1-IN-1), **(I).** PF-543, **(J).**SK1-I, **(K).** DHS (Safingol), **(L)**DMS, **(M)** K145, **(N)** SG12, **(O)**SG14, **(P)**Amgen 82, **(Q)** FTY720, **(R)** (S)-FTY720 vinyl phosphonate, **(S)** ROMe (R)-FTY720-OMe) showing their interaction with the key amino acids in the binding site for SPK inhibitors with the active site of Sphingosine Kinase 1 (3VZB). The figure was drawn by using MOE.2015.

| **(A)**  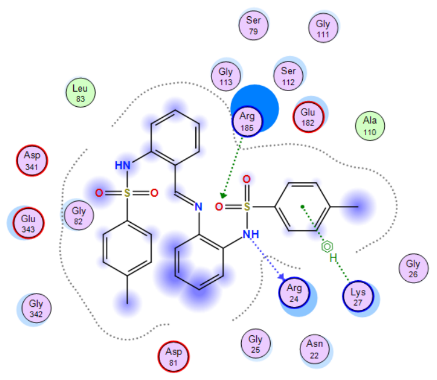 | **(B)**  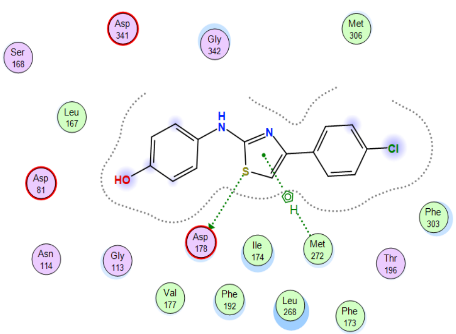 | **(C)**  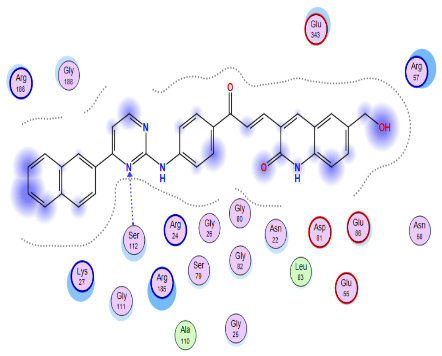 |
| --- | --- | --- |
| **(D)**  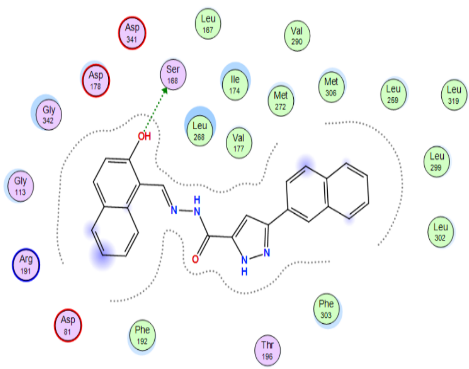 | **(E)**  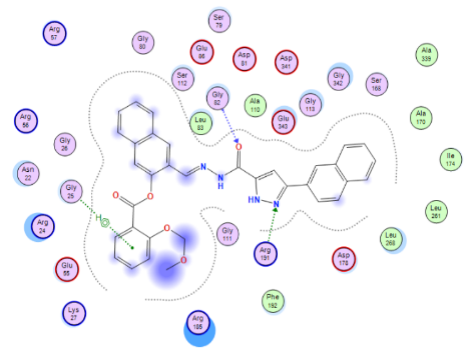 | **(F)**  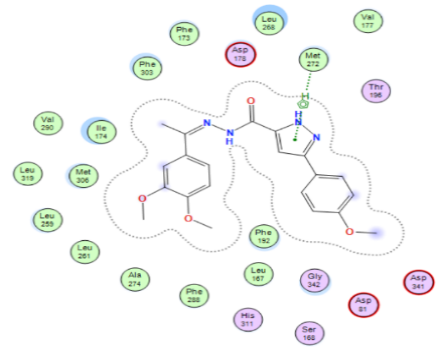 |
| **(G)**  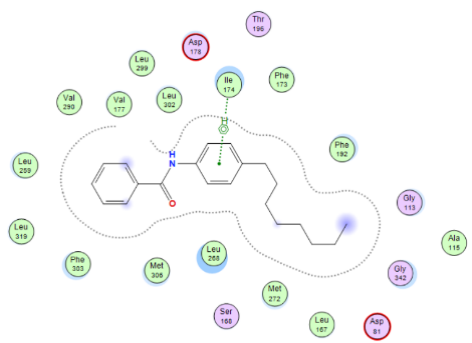 | **(H)**  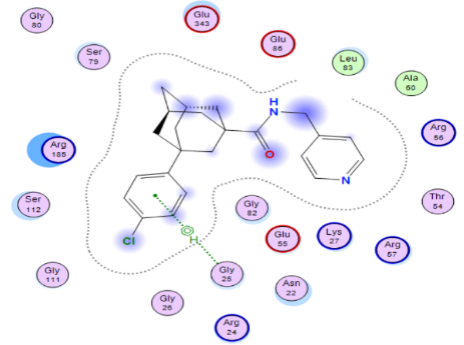 | **(I)**  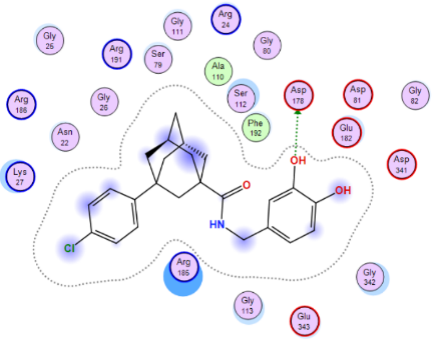 |
| **(J)**  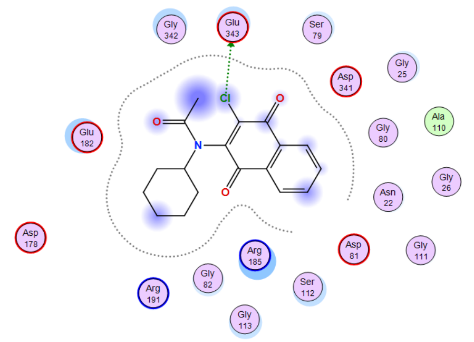 | **(K)**  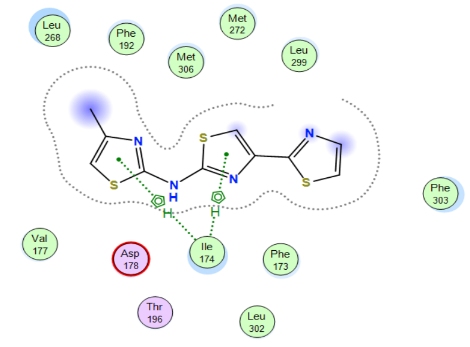 |  |

**Fig. S2**. 2D diagrams of the reported inhibitors **(A)**. MP-A08, **(B).** SKI-II, **(C).** 11b, **(D).** SKI-I, **(E).**  SKI-I-Asp, **(F).** SKI-178, **(G).** SK-F, (**H).** Opaganib ABC294640, **(I).** ABC294735, **(J).** CB5468139, **(K).** ST-1803 shows their interaction with the key amino acids in the binding site for SPK inhibitors with the active site of Sphingosine Kinase 1 (3VZB). The figure was drawn by using MOE.2015.

| **(A)**  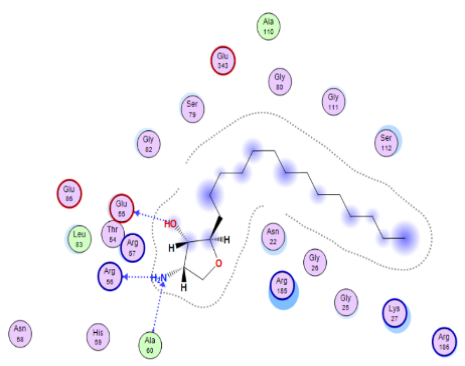 | **(B)**  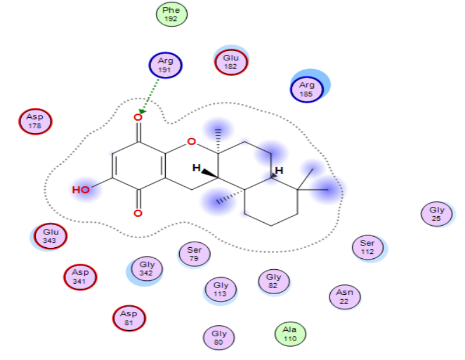 | **(C)**  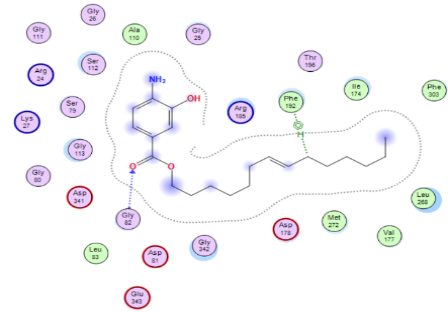 |
| --- | --- | --- |
| **(D)**  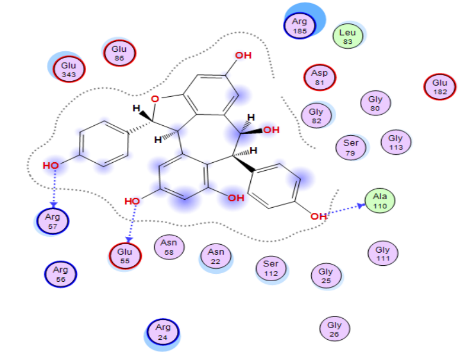 | **(E)**  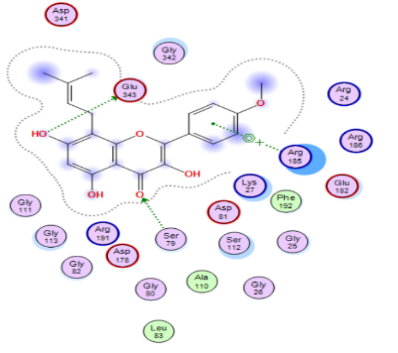 | **(F)**  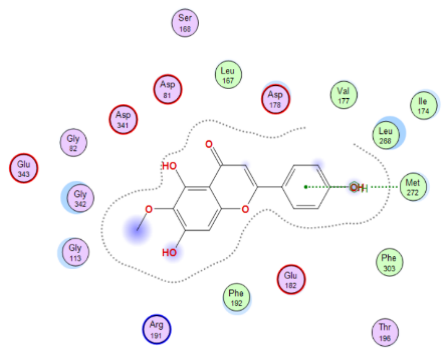 |
| **(G)**  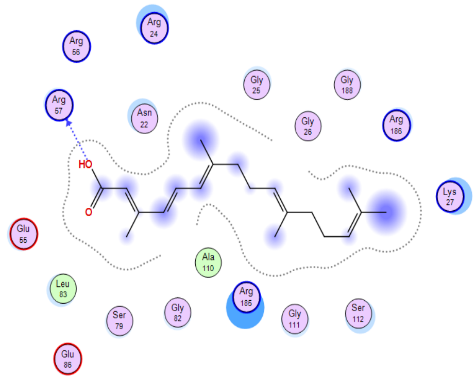 | **(H)**  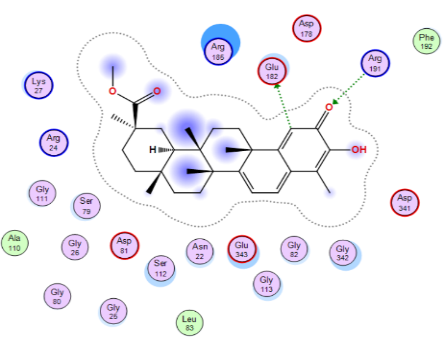 | **(I)**  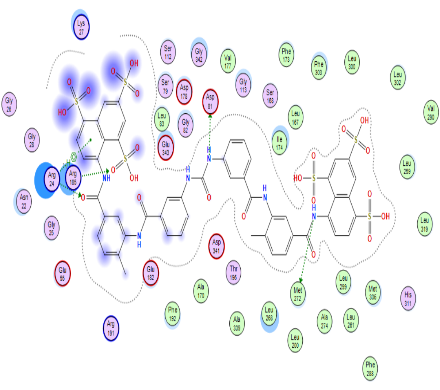 |
| **(J)**  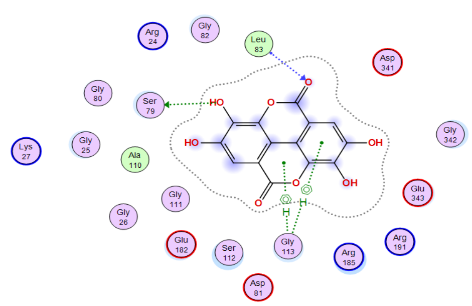 | **(K)**  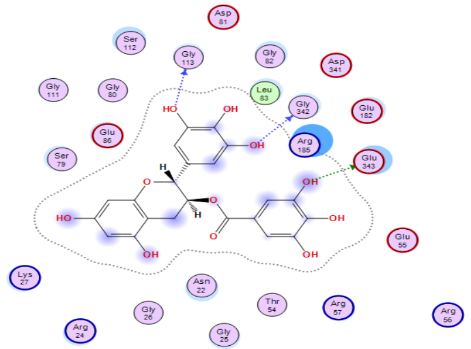 |  |

**Fig. S3. 2D** diagrams of the reported inhibitors **(A)**. Pachastrissamine (jaspine B), **(B)**. F-12509a, **(C)**. B-5354C, **(D)**. Balanocarpol, **(E)**. Icaritin, **(F)**. Hispidulin, **(G)**. Peretinoin, **(H)**. Pristimerin, **(I).** Suramin, **(J)**. Ellagic acid (EA), **(K)**. Epigallocatechin-3-gallate (EGCG) shows their interaction with the key amino acids in the binding site for SPK inhibitors with the active site of Sphingosine Kinase 1 (3VZB). The figure was drawn by using MOE.2015.
